# Melatonin Enhances the Usefulness of Ionizing Radiation: Involving the Regulation of Different Steps of the Angiogenic Process

**DOI:** 10.3389/fphys.2019.00879

**Published:** 2019-07-11

**Authors:** Alicia González-González, Alicia González, Noemí Rueda, Carolina Alonso-González, Javier Menéndez-Menéndez, José Gómez-Arozamena, Carlos Martínez-Campa, Samuel Cos

**Affiliations:** ^1^Department of Physiology and Pharmacology, School of Medicine, University of Cantabria and Instituto de Investigación Sanitaria Valdecilla (IDIVAL), Santander, Spain; ^2^Department of Medical Physics, School of Medicine, University of Cantabria, Santander, Spain

**Keywords:** melatonin, endothelial cells, radiation, HUVEC, aromatase

## Abstract

Radiotherapy is a part of cancer treatment. To improve its efficacy has been combined with radiosensitizers such as antiangiogenic agents. Among the mechanisms of the antitumor action of melatonin are antiangiogenic effects. Our goal was to investigate whether melatonin may modulate the sensitivity of endothelial cells (HUVECs) to ionizing radiation. Melatonin (1 mM) enhanced the inhibition induced by radiation on different steps of the angiogenic process, cell proliferation, migration, and tubular network formation. In relation with the activity and expression of enzymes implicated in estrogen synthesis, in co-cultures HUVECs/MCF-7, radiation down-regulated aromatase mRNA expression, aromatase endothelial-specific promoter I.7, sulfatase activity and expression and 17β-HSD1 activity and expression and melatonin enhanced these effects. Radiation and melatonin induced a significant decrease in VEGF, ANG-1, and ANG-2 mRNA expression. In ANG-2 and VEGF mRNA expression melatonin potentiated the inhibitory effect induced by radiation. In addition, melatonin counteracted the stimulatory effect of radiation on FGFR3, TGFα, JAG1, IGF-1, and KDR mRNA expression and reduced ANPEP expression. In relation with extracellular matrix molecules, radiation increased MMP14 mRNA expression and melatonin counteracted the stimulatory effect of radiation on MMP14 mRNA expression and increased TIMP1 expression, an angiogenesis inhibitor. Melatonin also counteracted the stimulatory effect of radiation on CXCL6, CCL2, ERK1, ERK2, and AKT1 mRNA expression and increased the inhibitory effect of radiation on NOS3 expression. In CAM assay, melatonin enhanced the reduction of the vascular area induced by radiation. Melatonin potentiated the inhibitory effect on the activation of p-AKT and p-ERK exerted by radiation. Antiangiogenic effect of melatonin could be mediated through AKT and ERK pathways, proteins involved in vascular endothelial (VE) cell growth, cell proliferation, survival, migration, and angiogenesis. In addition, radiation increased endothelial cell permeability and melatonin counteracted it by regulating the internalization of VE-cadherin. Radiation has some side effects on angiogenesis that may reduce its effectiveness against tumor growth and melatonin is able to neutralize these negative actions of radiation. Additionally, melatonin potentiated radiation-induced antiangiogenic actions on several steps of the angiogenic process and enhanced its antitumor action. Our findings point to melatonin as a useful molecule as adjuvant to radiotherapy in cancer treatment.

## Introduction

The generation of new blood vessels is a fundamental phenomenon that can be induced by pathological conditions as cancer. Angiogenesis needs degradation of the vascular basement membrane and the activation of endothelial cell proliferation, migration and tube formation (Bergers and Benjamin, 2003). Many studies suggest that inhibition of angiogenesis can reduce the development of tumors (Bareschino et al., 2011; Viallard and Larrivée, 2017). When the equilibrium between pro- and anti-angiogenic factors is altered, the “angiogenic switch” is activated. These factors, combined with endothelial cells, the extracellular matrix and tumor cells modulate endothelial cell activities and vessel stabilization (Bareschino et al., 2011; Dvorak et al., 2011; Viallard and Larrivée, 2017). Vascular endothelial growth factor (VEGF) and angiopoietins are the major pro-angiogenic factors (Cook and Figg, 2010; Danza et al., 2013). In angiogenesis a quantitatively and temporally coordinated dynamic equilibrium between the synthesis of VEGF and angiopoietins is needed (Bhadada et al., 2011).

Melatonin has oncostatic actions in diverse cancer types, especially hormone-dependent tumors (Hill and Blask, 1998; Cos and Sánchez-Barceló, 2000). Different mechanisms of action explain melatonin’s ability to counteract cancer genesis and growth. These involve indirect and direct antiestrogenic actions, induction of apoptosis, antioxidant effects, epigenetic effects, activation of the immunity, prevention of circadian disruption, inhibition of telomerase activity, regulation of tumor metabolism, and inhibition of angiogenesis (Cos et al., 1991; Molis et al., 1994; Blask et al., 2003; González et al., 2007, 2012; Mediavilla et al., 2010; Alvarez-García et al., 2013a,b,c; Reiter et al., 2017). Melatonin antiangiogenic effects are exerted directly on several important proteins in this process such as VEGF and angiopoietins (ANG-1 and ANG-2), by down-regulating VEGF expression in breast cancer cells and reducing the proliferation as well as the migration of endothelial cells, disrupting tube formation and counteracting the VEGF-stimulated capillary network formation (Alvarez-García et al., 2013a,b; Cos et al., 2014; González-González et al., 2018). Melatonin also shows indirect antiangiogenic effects, such as the inhibition of some tumor growth factors (IGF, EGF, and ET-1) which stimulate tumoral angiogenesis (Kajdaniuk et al., 2002) and the neutralization of reactive oxygen species which, in hypoxia, stabilize hypoxia-inducible factor HIF-α (Fandrey and Genius, 2000).

Radiation therapy plays a main role in cancer treatment. Although radiation has important positive effects in the treatment of many tumors, there are acute and chronic side effects that limit its use and affect the quality of life of patients. For this reason, different radioprotective compounds, such as antioxidants, corticosteroids or compounds containing thiol/sulfhydryl/SH, and radiosensitizers have been used with the aim of reducing these adverse effects in healthy tissue and improving the tumor response to radiotherapy treatment (Bagheri et al., 2018; Narmani et al., 2018). In order to bettering radiotherapy efficacy there is an increasing interest in combined radiotherapy with other therapies such as antiangiogenic therapy or immunotherapy (Goedegebuure et al., 2019).

Melatonin is a natural compound regulator of malignant cell growth that could have enhancing actions on the antineoplastic effects of radiation therapy (Farhood et al., 2019). Different *in vitro* and *in vivo* studies described that melatonin could behave as radioprotector. They have showed the ability of melatonin to reduce cytotoxic effects of ionizing radiation, such as DNA damage, apoptosis, fibrosis, inflammation, infertility, cataract, etc. (Vijayalaxmi et al., 2004). In addition, melatonin is also able to increase the oncostatic effects of radiation therapy. Both melatonin radioprotective and radiosensitive effects make it a good candidate to employ in combination to radiotherapy. Although studies on the mechanisms of the radiosensitive effects of melatonin are very limited, it has been suggested that the reduction of the DNA repair response, changes in the metabolism of the cancer cell, changes in estrogen biosynthesis, immunomodulatory actions or activation of proapoptotic proteins such as p53, could be some of the mechanisms involved in this process (Vijayalaxmi et al., 2004; Najafi et al., 2017; Farhood et al., 2019). Since melatonin has oncostatic actions in human breast cancer, part of these antitumor actions are aimed to prevent the formation of new vessels and also has beneficial effects added with ionizing radiation, our objective in the present study was to investigate the usefulness of melatonin as adjuvant to ionizing radiation in different steps of the angiogenic process. We analyzed the possible effects of melatonin on angiogenesis in radiated human endothelial cells in order to study whether this indolamine potentiates antiangiogenic actions and/or neutralizes proangiogenic actions induced by the radiation. To accomplish this, firstly, in radiated human endothelial cells, we studied melatonin effects on cell proliferation, migration of endothelial cells, formation of tubular structures, endothelial cell permeability, activity and expression of enzymes involved in estrogen biosynthesis, VEGF, and angiopoietins gene expression (two of the main pro-angiogenic factors). Secondly, we analyzed the influence of melatonin on the expression of different genes involved in biological processes of angiogenesis, by using a Human Angiogenesis RT^2^ Profiler^TM^ PCR Array. Those genes which mRNA expression was significantly regulated by melatonin were analized by specific RT-PCR studies. In addition, we explored some intracellular signaling pathways involved in melatonin effects. Finally, we evaluated melatonin anti-angiogenic activity *in vivo* on the chick embryo chorioallantoic membrane.

## Materials and Methods

### Cells and Culture Conditions

Human umbilical vein endothelial cells (HUVECs) were obtained from the American Tissue Culture Collection (Rockville, MD, United States). They were maintained as monolayer cultures in 58, 1 cm^2^ plastic culture plates in Vascular Cell Basal Medium (VCBM) (ATCC, Rockville, MD, United States) supplemented with endothelial cell growth kit-BBE (ATCC, Rockville, MD, United States) which consisted on 2% fetal bovine serum (FBS), 0.2% Bovine Brain Extract, 5 ng/ml rhEGF, 10 mM L-glutamine, 0.75 units/ml heparin sulfate, 1 μg/ml hydrocortisone hemisuccinate, 50 μg/ml ascorbic acid, penicillin (20 units/ml), and streptomycin (20 μg/ml) (Sigma-Aldrich, Madrid, Spain) at 37° in a humid atmosphere containing 5% CO_2_. To avoid genetic mutation and low viability, no more than five-six passages of HUVECs were used (Alvarez-García et al., 2013b,c).

MCF-7 human breast cancer cells were purchased from the American Tissue Culture Collection (Rockville, MD, United States). They were maintained as monolayer cultures in 75 cm^2^ plastic culture flasks in Dulbecco’s Modified Eagle’s Medium (DMEM) (Sigma-Aldrich, Madrid, Spain) supplemented with 10% FBS (PAA Laboratories, Pasching, Austria), penicillin (20 units/ml) and streptomycin (20 μg/ml) (Sigma-Aldrich, Madrid, Spain) at 37° in a humid atmosphere containing 5% CO_2_ (Alvarez-García et al., 2013a).

### Co-culture of HUVEC and MCF-7 Cells

Since it is known that the presence of malignant epithelial cells in the culture enhances estrogen formation in endothelial cells (Meng et al., 2001; Alvarez-García et al., 2012), in some experiments we employed co-cultures of HUVECs and MCF-7. Based on previous works (González-González et al., 2018) cells were co-cultured using Falcon 6-multiwell plates and Falcon cell culture inserts. HUVECs, which have been treated for a week with melatonin 1 mM or its diluent, were plated (50 × 10^4^ cells/well) on the bottom wells in VCBM supplemented with 2% FBS and incubated overnight. At the same time, MCF-7 cells (40 × 10^4^ cells), which have been treated for a week with melatonin 1 mM or its diluent, were cultured on the permeable membrane (0.45 μm) of the tissue-culture inserts in DMEM supplemented with 10% FBS also for 24 h. Both types of cells were cultured independently for 24 h. Then, MCF-7 seeded inserts were moved over HUVECs cell cultures in the 6-well plates in fresh VCBM supplemented with 2% FBS to create the hanging co-culture setup. Cell to cell contact is avoided but paracrine signaling take place between HUVECs in the well and MCF-7 cells on the insert. After 24 h, media were changed to VCBM supplemented with 2% FBS or serum free, according to the experiment. Then, cells were irradiated and incubated for 4 h to measure specific mRNA gene expression or for 0.5 or 24 h to measure the different enzymatic activities in HUVECs.

### Ionizing Radiation Treatment

HUVEC and MCF-7 cells were exposed to X irradiation using a model YXLON SMART 200 tube (Yxlon International, Hamburg, Germany) at room temperature. Initially, we used different doses, 4, 6, 8, and 10 Gy, of radiation. For the rest of experiments, as in previous works (Alonso-González et al., 2016), we used 8 Gy radiation as the optimal radiation dose. The radiation was administered as a single dose of 8 Gy in an 11.5 cm × 8.5 cm field size, 6-multiwell plates, at a dose rate of 0.92 Gy/min. The source-half-depth distance was initially calculated to obtain a constant dose rate of 0.92 Gy/min. Control cells were removed from the incubator and were place for the same period of time into the irradiator but without radiation.

### Measurement of Cellular Proliferation

Since the reduction of tetrazolium salts is accepted as a good way to study cell proliferation, we used the MTT [3(4,5dimethylthiazol-2-yl)-2,5-diphenyl tetrazolium bromide] method (Mosmann, 1983), as previously described (Alonso-González et al., 2016). MTT was obtained from Molecular Probes Inc. (Eugene, OR, United States). Cells were incubated for 1 week in VCBM supplemented with 2% FBS containing melatonin (1 mM) (Sigma-Aldrich, Madrid, Spain) or not. Then, both melatonin pretreated and control cells were seeded into 96-multiwell plates at a density of 8 × 10^3^ cells per well, and incubated at 37°C for 24 h. After irradiation cells were cultured for 3 days and cell proliferation was measured reading absorbance at 570 nm in a microplate reader (Labsystems Multiskan RC 351, Vienna, VA, United States).

### Measurement of Cellular Aromatase Activity

Aromatase activity in HUVECs cells was measured by the tritiated water release assay which is based on the formation of tritiated water during aromatization of a labeled androgenic substrate such as [1β-3H(N)]-androst-4-ene-3,17-dione (Ackerman et al., 1981). HUVECs, which have been treated for a week with melatonin 1 mM or its diluent, were co-cultured with MCF-7 cells as indicated previously. Then, when a homogenous monolayer of pre-confluent HUVECs was reached, media were aspirated and changed to serum-free media containing the labeled substrate 300 nM [1β-3H(N)]-androst-4-ene-3,17-dione (NEN Life Science Products, Boston, MA, United States) (75–80 Ci/nM) and cells were irradiated. After 24 h of irradiation, as we previously described (Alonso-González et al., 2016), culture flasks were placed on ice for 15 min to condense any water vapor and media were transferred to tubes containing 0.25 ml ice-cold 30% tricholoroacetic acid (w/v), vortexed and centrifuged at 1700 *g* for 15 min at 4°C. The supernatants were extracted with chloroform, vortexed and centrifuged at 1700 *g* for 15 min at 4°C. The resulting aqueous supernatants were adsorbed with 5% dextran-coated charcoal (Sigma-Aldrich, Madrid, Spain), vortexed, centrifuged at 1700 *g* for 15 min at 4°C and the supernatants were added to vials with scintillation cocktail and counted in a beta counter (Beckman LS 6000 IC, Fulleton, CA, United States). The amount of radioactivity measured in [^3^H]-water was corrected by substracting the blank values from each sample, obtained by incubating dishes containing medium with the tritiated androgen but no cells. The values were also corrected by taking into account the fractional retention of tritium in medium water throughout the processing, utilizing parallel dishes containing medium plus known amounts of [^3^H]-water (NEN Life Science Products, Boston, MA, United States) through incubation and assay. The fractional retention of tritium in medium water throughout the incubation and processing of samples was always higher than 85%.

### Measurement of Steroid Sulfatase (STS) Activity

STS activity in HUVECs was analyzed by the formation of estrone from a labeled substrate ([6,7-^3^H(N)]-estrone sulfate ammonium salt) (Duncan et al., 1993). HUVECs, which have been treated for a week with melatonin 1 mM or its diluent, were co-cultured with MCF-7 cells as indicated previously. As we previously described (Alonso-González et al., 2016), when a homogenous monolayer of pre-confluent HUVECs was reached, media were replaced with fresh ones (1 ml per plate) containing 20 nM [6,7-^3^H(N)]-estrone sulfate ammonium salt (NEN Life Science Products, Boston, MA, United States) (57.3 Ci / mM) and cells were irradiated. After 24 h of irradiation, culture dishes were processed as earlier described (Alonso-González et al., 2016).

### Measurement of 17β-Hydroxysteroid Dehydrogenase Type 1 (17β-HSD1) Activity

Activity of 17β-HSD1 was studied in endothelial cells by the formation of estradiol from a labeled substrate [2,4,6,7-^3^H(N)]-estrone (Singh and Reed, 1991). HUVECs, which have been treated for a week with melatonin 1 mM or its diluent, were co-cultured with MCF-7 cells as indicated previously. Then, when a homogenous monolayer of pre-confluent HUVECs was reached, media were aspirated, changed for fresh ones (1 ml per plate) containing 2 nM [2,4,6,7-^3^H(N)]-estrone (NEN Life Science Products, Boston, MA, United States) (100 Ci / mM) and cells were irradiated. After 30 min of incubation, the media were processed as we previously described (Alonso-González et al., 2016).

### Human Angiogenesis RT^2^ Profiler^TM^ PCR Array

A 96-well Human Angiogenesis RT^2^ Profiler^TM^ PCR Array (Qiagen, United States) was used to perform a pathway-focused gene expression profiling. Each well of the array contains all the components required and designed to generate single, gene-specific amplicons, testing the expression of 84 genes related to angiogenesis (growth factors and receptors, adhesion molecules, proteases, inhibitors and other matrix proteins, transcription factors, cytokines, and chemokines), and five housekeeping genes. Briefly, HUVECs, which have been treated for a week with melatonin 1 mM or its diluent, were seeded into 60 × 15 mm plates in VCBM supplemented with 2% FBS and incubated at 37°C for 24 h to allow for cell attachment. Then media were replaced by fresh ones with 2% FBS and plates were irradiated. After 4 h of incubation, total RNA was extracted and reverse transcribed. The cDNA template was combined with the appropriate amount of RT^2^ SYBR Green qPCR Mastermix (Qiagen GmbH, Germany), aliquoted 25 μl to each well of the same plate, and carried out the real-time PCR cycling program in an MX3005P (Agilent, CA, United States) according to the manufacturer’s instructions. Amplification was initiated by 1 cycle at 95°C for 10 min and then performed for 40 cycles for quantitative analysis using the following temperature profile: 95°C for 30 s (denaturation); 60°C for 60 s (annealing/extension). Dissociation curves were made to confirm that only a single product was amplified. The Ct data for each gene were analyzed using the Qiagen RT^2^ profiler PCR array data analysis software. Data are showed as fold-regulation between the experimental groups and the control cells. Fold-change values less than one indicate a negative or downregulation, and the fold-regulation is the negative inverse of the fold-change.

### Measurement of Specific mRNA Gene Expression

Analysis of different genes mRNA expression in HUVECs were carried out by real-time reverse transcription RT-PCR after cells, which have been treated for a week with melatonin 1 mM or its diluent, were irradiated and incubated for 4 h. The total cellular RNA was isolated from HUVECs and purified with the Nucleospin RNA II Kit (Machenery-Nagel, Düren, Germany) following the manufacturer’s instructions. As we previously described (Alonso-González et al., 2016), integrity of RNA was assessed by electrophoresis in ethidium bromide-stained 1% agarose-Tris-borate EDTA gels. The absorbance ratio A_260__nm_/A_280__nm_ was >1.8. For cDNA synthesis 0.5 μg of total RNA was denatured at 65°C for 10 min and reverse transcribed for 50 min at 45°C with cDNA Synthesis kit (Bioline, London, United Kingdom) in a final volume of 20 μl in the presence of 500 ng of oligo (dT) 12–18 primers. The primers used for amplification (Sigma Genosys Ltd., Cambridge, United Kingdom) are described in Table 1. As a control quantification, s14 mRNA was used (Sigma Genosys Ltd., Cambridge, United Kingdom). RT-PCRs were performed in a MX3005P system (Stratagene, La Jolla, CA, United States) using Brilliant^®^ SYBR^®^ Green PCR Master Mix (Applied Biosystems, Madrid, Spain) following the manufacturer’s instructions. Amplifications were performed for 40 cycles using the following temperature profile: 60°C, 45 s (annealing); 72°C, 30 s (extension); and 95°C, 30 s (denaturation). Melting curves were performed to confirm that only a single product with no primer-dimers was amplified. For the primers used there were no differences between transcription efficiencies, and the fold-change in each sample was calculated by the 2^-ΔΔ^Ct method (Livak and Schmittgen, 2001).

**Table 1:** Primers used for amplification of mRNA transcripts.

Human gene	Sense primer (5′-3′)	Antisense primer (5′-3′)
17β-HSD1	CTTATGCGAGAGTCTGGCGGTTC	GGTATTGGTAGAAGCGGTGGAAGG
AKT1	AAGTACTCTTTCCAGACCC	TTCTCCAGCTTGAGGTC
ANG1	GAAGGGAACCGAGCCTATTC	AGGGCACATTTGCACATACA
ANG2	AAGAGAAAGATCAGCTACAGG	CCTTAGAGTTTGATGTGGAC
ANPEP	CCTTCATTGTCAGTGAGTTC	CAGCAAAGAAGTTAAGGATGG
ARO	GTCGTGGACTTGGTCATGC	CGAGTCTGTGCATCCTTCC
CCL2	AGACTAACCCAGAAACATCC	ATTGATTGCATCTGGCTG
CXCL6	CCTCTCTTGACCACTATGAG	GTTTTGGGGTTTACTCTCAG
ERK1	GCCACCTTCTCTCACTTTGCTG	CCCACATCCAATCACCCACA
ERK2	CAGCACCTCAGCAATGACCA	TGCGGGAGAGAAAGCAAATAGT
FGFR3	GAAGATGCTGAAAGACGATG	GCAGGTTGATGATGTTTTTG
IGF1	CCCAGAAGGAAGTACATTTG	GTTTAACAGGTAACTCGTGC
JAG1	ACTACTACTATGGCTTTGGC	ATAGCTCTGTTACATTCGGG
KDR	GTACATAGTTGTCGTTGTAGG	TCAATCCCCACATTTAGTTC
MMP-14	GATAAACCCAAAAACCCCAC	CTCCTTGAAGACAAACATCTC
NOS3	CAACCCCAAGACCTACG	CGCAGACAAACATGTGG
pI.7	AACACTCAGCTTTTTCCCAAC	CTTGCTGATTTCACCCCTTT
STS	TCCGTTCCTGCTTGTCTTGTC	CCTGGTCCGATGTGAAGTAGATG
TGFα	AGAAACAGTGGTCTGAAGAG	ATTACAGGCCAAGTAGGAAG
TIMP1	CACCTTATACCAGCGTTATG	TTTCCAGCAATGAGAAACTC
VEGFB	GAAAGTGGTGTCATGGATAG	ATGAGCTCCACAGTCAAG
S14	TCACCGCCCTACACATCAAAC	TCCTGCGAGTGCTGTCAGAG


### Western Blot Analysis: Immunodetection of Protein Expression Levels of AKT, pAKT, ERK, and pERK

Confluent cells were washed with PBS and proteins were collected using RIPA buffer, containing 1% protease inhibitor cocktail. The protein concentration was determined using the method of Lowry (Sengupta and Chattopadhyay, 1993) in the spectrophotometer (Multiskan ex, Thermo Scientific) at a wavelength of 620 nm, using a standard curve of albumin. The cellular proteins were then separated by SDS-PAGE and transferred to a polyvinylidene fluoride (PVDF) membrane (Bio-Rad) from the polyacrylamide gel. The membranes were blocked by incubation (1 h at room temperature) in TBS-T buffer (10 mM Tris–HCl, pH 7.6, 150 mM NaCl, and 0.05% Tween 20) with 3% bovine serum albumin (BSA). Subsequently the membranes were incubated with the primary antibody, first for 1 h at room temperature and then overnight at 4°C and under stirring, and then incubated with the respective fluorescent secondary antibody for 30 min at room temperature. The following antibodies were used: rabbit anti-AKT antibody (Cell Signaling Technology, Danvers, MA, United States), rabbit anti-P-AKT antibody (Santa Cruz, CA, United States), mouse anti-ERK½ antibody (Santa Cruz, CA, United States), rabbit anti-P-ERK½ antibody (Cell Signaling Technology, Danvers, MA, United States), and mouse anti-GAPDH antibody (Santa Cruz, CA, United States). Protein bands were detected by incubating with anti-rabbit IRDye- 680RD LI-COR Biotechnology, Lincoln, Nebraska, United States Red (700 nm) or anti-mouse IRDye-800CW LI-COR Biotechnology, Lincoln, Nebraska, United States Green (800 nm). Fluorescence was detected using the LI-COR Odyssey IR Imaging System V3.0 system (LI-COR Odyssey Biosciences). The optical density of the bands was determined with the ImageJ program.

### Endothelial Cell Migration Assay: Wound Healing Assay

Endothelial cell migration was studied as previously described (Alvarez-García et al., 2013a). Briefly, HUVEC control or melatonin treated for a week cells were cultured into 6-well plates (Falcon) in VCBM supplemented with 2% FBS and were allowed to reach full confluence. With a pipette tip a line of cells was scraped away. Then, cells were washed with PBS and irradiated. In the plates, four randomly selected views along the scraped line were photographed using an ORCA R2 camera (Hamamatsu Photonics, Massy Cedex, France) attached to a microscope set Nikon Ti (Werfen Group, Barcelona, Spain) at 10 × magnification. Photomicrographs were taken every 10 min during the course of the experiment, which was terminated as soon as the wound was completely filled in vehicle treated controls (after 10 h). Initial and final wound sizes were measured using the Nis Elements v.3.8 software (Nikon, Tokyo, Japan) and the difference between the two was used to determine migration distance using the following formula: initial wound size minus final wound size divided by two.

### Endothelial Cell Differentiation Assay: Endothelial Cell Capillary-Like Tube Formation Assay

To study the formation of tubular structures in HUVECs we used the *in vitro* Angiogenesis Assay Tube Formation Kit (Cultrex, Trevigen Inc., Gaithersburg, MD, United States) according to the supplier’s instructions, as previously described (Alvarez-García et al., 2013a). Briefly, growth factor-reduced Basement Membrane Extract (BME) was added to a 24-well plate and polymerized for 1 h at 37°C. Control and melatonin pretreated cells for a week (3 × 10^5^ per well) were then cultured in VCBM with 2% FBS and irradiated. Four h later, tubular structures in four random fields were photographed with a camera Nikon Sight DS-SML1 (Sendai Nikon Corporation, Miyagi, Japan) attached to a fluorescence microscope Nikon Eclipse TS100 (Kurobane Nikon Co Ltd., Tochigi, Japan) at 4×. The length of the tubular network was measured using ImageJ 1.45S software.

### Vascular Permeability Assay

Endothelial permeability was studied by diffusing of 40 kDa fluorescein isothiocyanate (FITC)-dextran through cells (Porcù et al., 2016). HUVECs control or melatonin treated for a week were grown to confluence on collagen-coated inserts. Then, to stimulate endothelial cell permeability VEGF 4 ng/ml was added to the plates and inserts and were radiated. After 24 h, FITC-dextran solution was added. We measured the fluorescence intensity of FITC-dextran that crossed the endothelial monolayer at 30, 60, and 90 min by using a microplate reader at 485/530 nm (Labsystems Multiskan RC 351, Vienna, VA, United States).

### Immunofluorescence Assay

Immunolabeling was performed in control or melatonin treated HUVECs grown on coverslips. Then, cells were irradiated and, after 24 h, fixed in 100% methanol (5 min) at room temperature. After washing carefully, crystals were placed in a Petri dish and, creating a moist chamber, cells were permeabilized with PBS contained 10% goat serum, 0.3 M glycine, 1% BSA and 0.1% tween for 1 h at room temperature. Primary antibody staining was performed for rabbit policlonal anti-VE-cadherin (ab33168) (Abcam, Cambridge, United Kingdom) at 1 μg/ml. After several PBS washes, cells were incubated for 1 h at room temperature with the secondary antibody conjugated with a fluorochrome, FITC (Jackson ImmunoResearch Laboratories, Inc., United States). After incubation, crystals were washed with 1× PBS, dried thoroughly and a drop of vectashield mounting medium (Vector Laboratories, Peterborough, United Kingdom) was added. Observation was made with a LSM51O laser confocal microscope (Zeiss).

### Chick Chorioallantoic Membrane (CAM) Model of Angiogenesis

As an *in vivo* model for studying angiogenesis we used the CAM assay as described elsewhere (Porcù et al., 2016). Fertilized eggs were incubated at 37°C in a humidified incubator for 3 days. Hypodermic needles were used to remove 4 ml of egg albumin to allow detachment of the developing CAM shell. At day 4, the shells were covered with a transparent adhesive tape and a small window sawed with scissors on the broad side directly over the avascular portion of the embryonic membrane. At day 11, eggs were irradiated at 3 Gy and alginate beads containing PBS, VEGF and/or melatonin 1 mM per bead were grafted on the CAM. VEGF 1.57 pM was used as a positive control whereas PBS was used as a negative control. Chemical agents were dissolved in 1% dimethyl sulfoxide. After 72 h new blood vessels converging toward the alginate were counted at 5 × magnification under a STEMI SR stereomicroscope equipped with a 100 mm objective with adapter ring 47070 (Zeiss) and fixed with 7% buffered formalin and photographed.

### Statistical Analysis

Statistical analyses were performed using GraphPad Prism software. The data are showed as the mean ± standard errors of the mean (S.E.M.). Statistical differences between groups were calculated by using one way analysis of variance (ANOVA), followed by the Student-Newman–Keuls test. Results were considered as statistically significant at *p* < 0.05.

## Results

### Influence of Melatonin on Ionizing Radiation-Induced Effects on Cell Proliferation in Endothelial Cells

**FIGURE 1:**
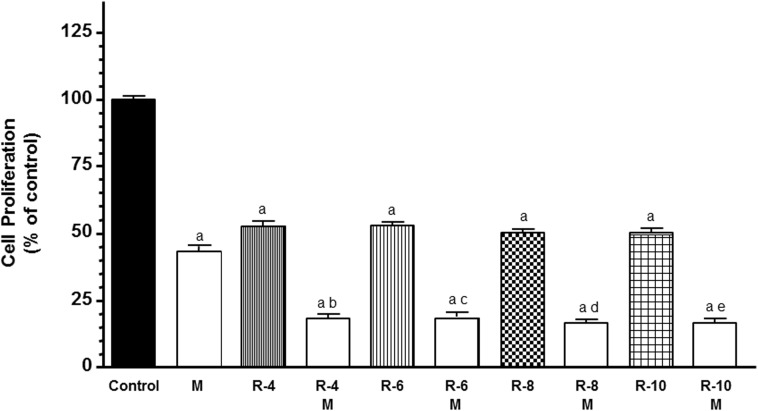
Melatonin increased radiation-induced effects on cell proliferation in endothelial cells. Cells were incubated in medium supplemented with 2% FBS containing melatonin 1 mM (M) or not for 1 week. At that time, both melatonin pretreated and control cells were seeded into 96-multiwell plates at a density of 8 × 10^3^ cells per well, and incubated at 37° for 24 h. Then, cells were irradiated with different doses, 4 (R-4), 6 (R-6), 8 (R-8), and 10 (R-10) Gy. After irradiation cells were cultures for 3 days and cell proliferation was measured by the MTT method. Data are expressed as the percentage of the control group (Mean ± SEM). a, *p* < 0.001 *vs* control; b, *p* < 0.001 *vs* R-4; c, *p* < 0.001 *vs* R-6; d, *p* < 0.001 *vs* R-8; and e, *p* < 0.001 *vs* R-10.

To study the influence of melatonin in the first step of angiogenic process, we analyzed the modulation of melatonin pretreatment on the antiproliferative effects of ionizing radiation in HUVECs. According with previous findings, melatonin pretreatment at 1 mM induced an inhibitory effect on the proliferation of endothelial cells (Figure 1). With radiation alone, the HUVECs proliferation decreased (*p* < 0.001) to all doses of radiation administered. Moreover, melatonin pretreatment for 7 days before radiation induced a significant (*p* < 0.001) higher decrease in cell proliferation in comparison with only radiated cells (Figure 1).

### Effects of Melatonin on Ionizing Radiation-Induced Changes on Endothelial Cell Migration

**FIGURE 2:**
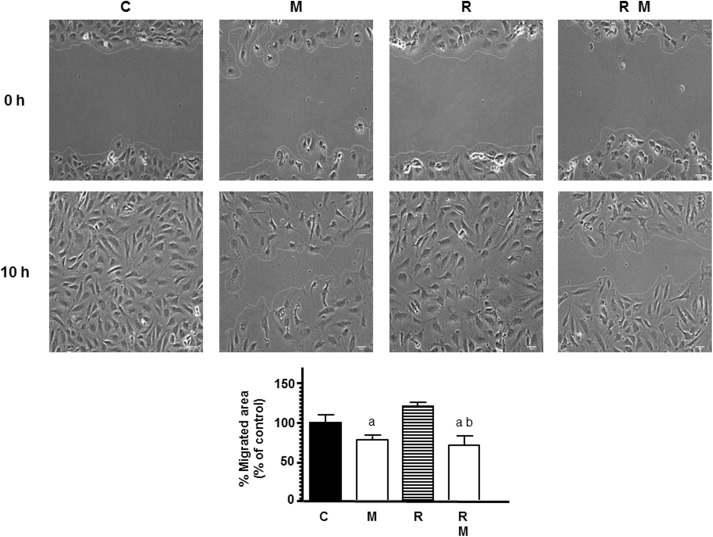
Effects of melatonin (M) and radiation (R) on endothelial cell migration analyzed through the wound healing assay. Representative photomicrographs of initial and after 10 h are shown at 10 × magnification and migration distance was measured as described in Material and Methods. Migrated area data shown are expressed as Mean ± SEM of four experiments. a, *p* < 0.05 *vs* C (control); b, *p* < 0.01 *vs* R.

To continue studying the angiogenic process, we next investigated the effects of melatonin pretreatment on the migratory properties of endothelial cells in radiated cells by using wound-healing assays. As showed in Figure 2, 8 Gy radiation alone did not modified the percentage of migrated endothelial cells in comparison to control cells. Melatonin pretreatment decreased the distance migrated by endothelial cells when compared to radiated cells alone.

### Effects of Melatonin on Ionizing Radiation-Induced Changes on HUVEC Capillary Structure Formation

**FIGURE 3:**
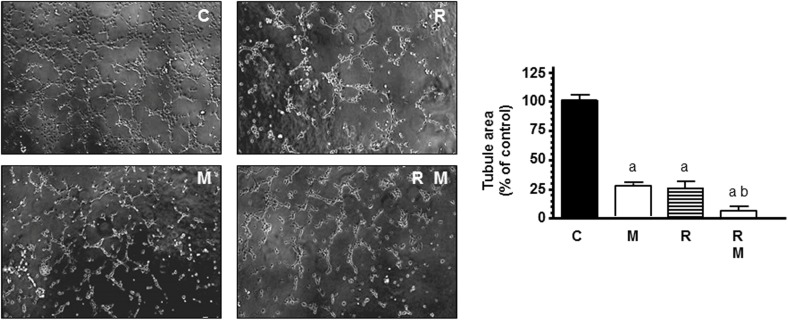
Potentiation by melatonin (M) of radiation (R)-induced effects on HUVEC capillary structure formation. HUVECs control (C) and melatonin pretreated cells for a week were seeded on growth factor-reduced BME in medium supplemented with 2% FBS and irradiated. After 4 h of incubation, tubular structures were photographed and tubule area was measured as described in see section “Material and Methods.” Tubule area data are expressed as the percentage of the control group (mean ± SEM). a, *p* < 0.001 *vs* C (control); and b, *p* < 0.05 *vs* R.

To mimic other step in the angiogenesis process, HUVECs were seeded on matrigel matrix to study the ability of endothelial cells to form a capillary network. Then, we studied whether ionizing radiation was able to interfere with the process of formation of tubes and the capacity of melatonin to modulate this action. As shown in Figure 3 endothelial cells plated on matrigel formed a large network of tubes after 4 h. Radiation significantly (*p* < 0.001) decreased the tubular network formation. In radiated cells, melatonin 1 mM pretreatment disrupted tube formation even more and increased significantly the reduction of tubule area induced by ionizing radiation.

### Influence of Melatonin on Ionizing Radiation-Induced Effects on Activity and Expression of Enzymes Involved in Local Estrogen Biosynthesis in Endothelial Cells

**FIGURE 4:**
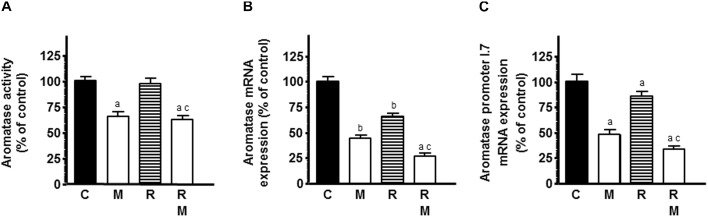
Effects of melatonin on radiation-induced changes on aromatase activity and expression and aromatase promoter 1.7 mRNA expression in endothelial cells. HUVECs treated for a week with melatonin 1 mM or its diluent were co-cultured with MCF-7 cells as indicated in see section “Material and Methods.” **(A)** When a homogenous monolayer of pre-confluent HUVECs was reached, media were aspirated and changed to serum-free media containing the labeled substrate 300 nM [1β-3H(N)]-androst-4-ene-3,17-dione and irradiated. After 24 h of incubation, aromatase activity was measured as described in material and methods. **(B,C)** Control and melatonin pretreated endothelial cells were irradiated and incubated for 4 h. The total cellular RNA was isolated from cells and reverse transcribed. cDNA was subjected to PCR using specific primers for aromatase and aromatase promoter 1.7 or S14. Data are expressed as the percentage of the control group (Mean ± SEM). C, control; M, melatonin; and R, radiation. a, *p* < 0.001 *vs* C (HUVEC); b, *p* < 0.05 *vs* C (HUVEC); c, *p* < 0.001 *vs* R (HUVEC); d, *p* < 0.05 *vs* C (HUVEC/MCF-7); e, *p* < 0.001 *vs* C (HUVEC/MCF-7); and f, *p* < 0.05 *vs* R (HUVEC/MCF-7).

The role of estrogens in the growth of breast cancer with pleiotropic effects on vascular tissue led us to study the modulation of melatonin on ionizing radiation-induced effects on activity and expression of several enzymes involved in local synthesis of estrogens in endothelial cells. Since our HUVEC cell line stock showed a low activity and expression of the enzymes implicated in estrogen synthesis and it is known that the presence of malignant epithelial cells in the culture enhances estrogen production in endothelial cells, we employed co-cultures of HUVECs and MCF-7 cells to improve the activity and expression of the enzymes. Firstly, we studied the influence of melatonin pretreatment on ionizing radiation-induced effects on aromatase activity in HUVECs incubated with tritiated androstenedione. Radiation did not modified the aromatase activity of HUVECs. However, melatonin pretreatment decreased significantly aromatase activity in the presence or not of radiation (Figure 4A). Then, we studied the ionizing radiation-induced effects on aromatase expression. The mRNA expression of aromatase was significantly reduced by radiation. Melatonin pretreatment at 1 mM exhibited a significant and potent decrease in aromatase mRNA expression and increased significantly the inhibitory effect induced by ionizing radiation (Figure 4B).

To determine whether the aromatase promoter mainly active in vascular endothelial (VE) cells in breast cancer, the aromatase-promoter I.7, is involved in the regulation of aromatase expression, we used RT-PCR to amplify the aromatase endothelial-specific promoter I.7 transcripts from RNA extracted from HUVECs. As shown in Figure 4C, radiation significantly downregulated the aromatase-promoter I.7 and melatonin enhanced this inhibitory effect.

**FIGURE 5:**
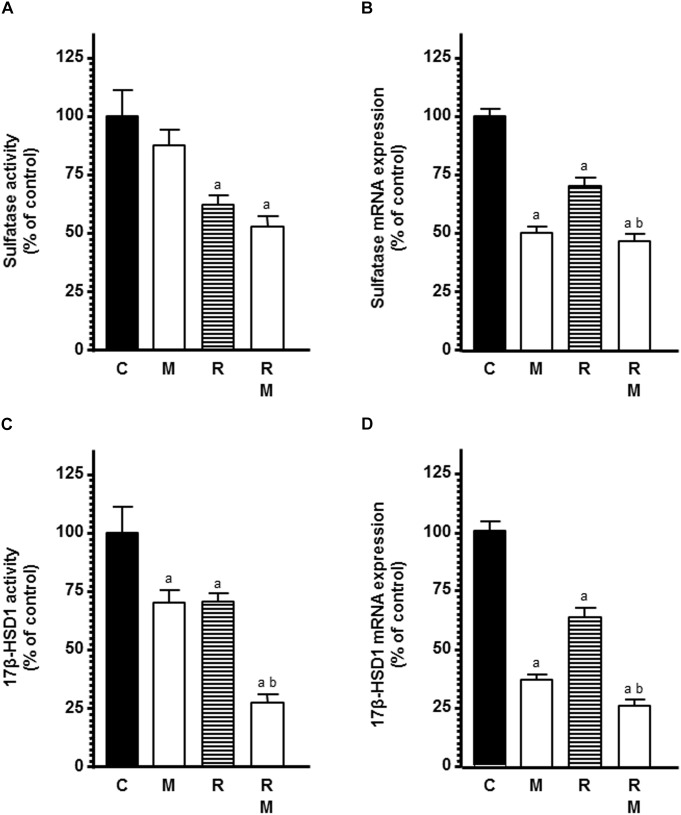
Effects of melatonin pretreatment on radiation-induced changes on STS and 17β-HSD1 activity and expression in endothelial cells. Melatonin 1 mM or its diluent pretreated endothelial and MCF-7 cells for a week were plated on the co-culture as described in material and methods. **(A)** When a homogenous monolayer of pre-confluent HUVECs was reached, media were aspirated and changed to serum-free media containing the labeled substrate 20 nM [6,7-^3^H(N)]-estrone sulfate ammonium salt and radiated. After 24 h of radiation, sulfatase activity was measured as described in material and methods. **(C)** When a homogenous monolayer of pre-confluent HUVECs was reached, media were aspirated and changed for fresh ones containing 2 nM [2,4,6,7-^3^H(N)]-estrone (100 Ci/mM) and cells were irradiated. After 30 min of incubation, 17β-HSD1 activity was measured as described in material and methods. **(B,D)** Control and melatonin pretreated endothelial cells were irradiated and incubated for 4 h. The total cellular RNA was isolated from cells and reverse transcribed. cDNA was subjected to PCR using specific primers for STS, 17β-HSD1 or S14. Data are expressed as the percentage of the control group (Mean ± SEM). C, control; M, melatonin; and R, radiation. a, *p* < 0.001 *vs* C; and b, *p* < 0.001 *vs* R.

In a second set of experiments, we studied the influence of melatonin on ionizing radiation-induced effects on STS activity and mRNA expression, the enzyme responsible for converting estrogen sulfates into estrone and estradiol. Radiation induced a significant inhibition of the STS activity and expression. Melatonin pretreatment induced a significant (*p* < 0.001) higher down-regulation in the STS activity and expression (Figure 5A,B).

Next, we assessed the influence of melatonin on ionizing radiation-induced effects on 17β-HSD1 activity and mRNA expression, the enzyme that catalyze the conversion of the relatively weak estrone into the more potent estradiol. Both radiation and melatonin pretreatment decreased 17β-HSD1 activity. Melatonin pretreatment enhanced the inhibitory effect induced by the radiation on 17β-HSD1 activity (Figure 5C). When we studied 17β-HSD1 mRNA expression in endothelial cells, we found that melatonin and radiation also reduced its expression. Melatonin also enhanced the inhibitory effect induced by the radiation (Figure 5D).

### Effects of Melatonin on Radiation-Induced Effects on mRNA Expression of Some of the Main Pro-angiogenic Factors, Such as VEGF and Angiopoietins

**FIGURE 6:**
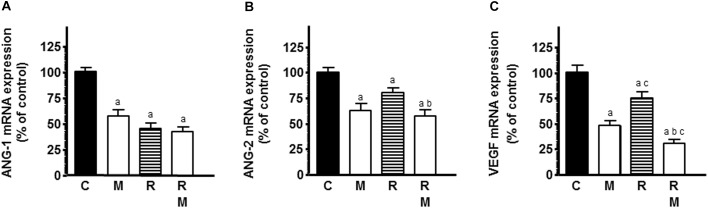
Effects of melatonin on radiation-induced changes on mRNA expression of some of the main pro-angiogenic factors, ANG-1 **(A)**, ANG-2 **(B)**, and VEGF **(C)**. HUVECs treated for a week with melatonin 1 mM or its diluent were co-cultured with MCF-7 cells, irradiated and incubated for 4 h, as indicated in material and methods. The total cellular RNA was isolated from cells and reverse transcribed. cDNA was subjected to PCR using specific primers for VEGF, ANG-1, ANG-2, or S14. Data are expressed as the percentage of the control group (mean ± SEM). C, control; M, melatonin; and R, radiation. a, *p* < 0.001 *vs* C; b, *p* < 0.001 *vs* R; and c, *p* < 0.05 *vs* M.

To study whether the effects in endothelial cell proliferation could be due to a downregulation of some of the main angiogenic factors, such as VEGF, ANG-1, and ANG-2, we measured ANG-1, ANG-2, and VEGF mRNA expression by RT-PCR. Radiation induced a significant decrease in ANG-1, ANG-2, and VEGF mRNA expression (Figure 6A–C). Melatonin had the same inhibitory effects as radiation. In ANG-2 and VEGF mRNA expression, melatonin pretreatment induced a significant downregulation of the expression and potentiated the inhibitory effect induced by radiation (Figure 6B,C).

### Effects of Melatonin on Changes Induced by Radiation on Gene Expression Related With Angiogenesis

**FIGURE 7:**
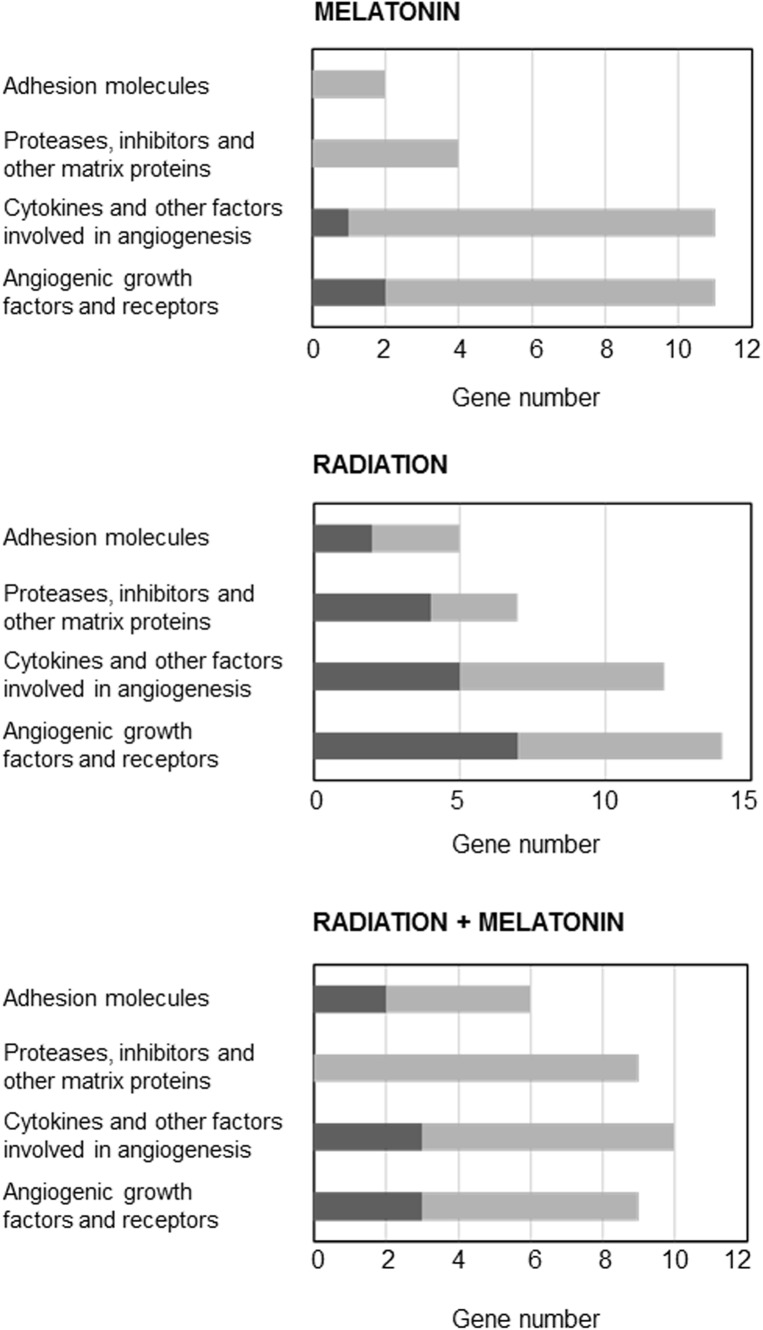
Genes induced or repressed at least two-fold in endothelial cells treated either with radiation and/or melatonin 1 mM for 4 h. Pathway-focused gene expression profiling was performed using a 96-well human breast cancer Human Angiogenesis RT^2^ Profiler^TM^ PCR Array. Black columns indicate the percentage of upregulated genes and gray columns indicate the percentage of downregulated genes in each category.

Since the changes induced by radiation on gene expression related with angiogenesis are quite unknown, we used the Human Angiogenesis RT^2^ Profiler^TM^ PCR Array to determine the changes induced by ionizing radiation either alone or in combination with melatonin (1 mM) on the gene expression of 84 genes related to angiogenesis. As shown in Figure 7, having as criteria a change of at least 2-fold either with radiation alone or in combination with melatonin (in comparison with the expression of untreated control cells), radiation upregulated the expression of 18 genes and downregulated the expression of 20 genes. Melatonin alone downregulated the expression of 25 genes and upregulated the expression of 3 genes. In combination with radiation melatonin induced a downregulation of the expression of 26 genes and an upregulation of the expression of 8 genes. Of all of them we chose those that had been altered in two or more array membranes, excluding those that we had previously studied (VEGF, ANG-1, and ANG-2) and those about which there is already a lot of information about its modulation by melatonin. Figure 7 shows the effect of radiation in the presence or not of melatonin on the most altered angiogenic factors.

**FIGURE 8:**
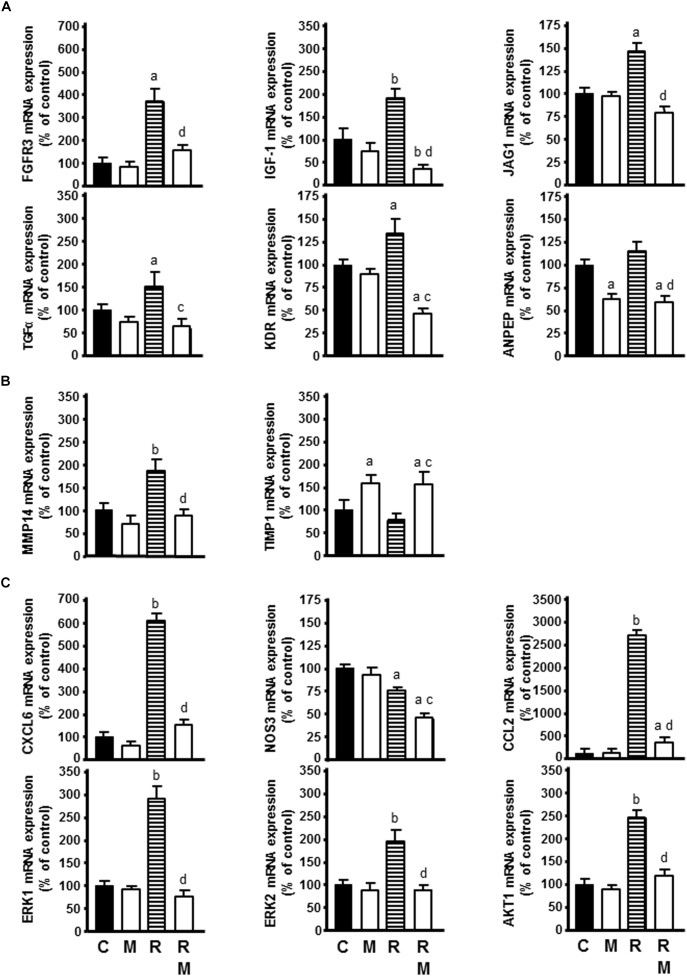
Effect of radiation in the presence or not of melatonin 1 mM on the genes selected for analysis by specific RT-PCR: **(A)** FGFR3, IGF-1, JAG1, TGFα, KDR, and ANPEP as angiogenic growth factors, **(B)** MMP14 and TIMP1 as extracellular matrix molecules, **(C)** CXCL6, NOS3, CCL2, ERK1, ERK2, and AKT1 as cytokines and other angiogenic factors. Data are expressed as the percentage of the control group (Mean ± SEM). C, control; M, melatonin; and R, radiation. a, *p* < 0.05 *vs* C; b, *p* < 0.001 *vs* C; c, *p* < 0.05 *vs* R; and d, *p* < 0.001 *vs* R.

Of the growth factors and receptors that behave as angiogenic factors, radiation increased FGFR3, IGF-1, JAG1, TGFα, and KDR mRNA expression. Melatonin counteracted this stimulatory effect of radiation and reduced ANPEP expression in comparison with only radiated cells (Figure 8A).

Of the proteases, inhibitors and other matrix proteins involved in angiogenesis, radiation increased MMP14 mRNA expression. Melatonin counteracted the stimulatory effect of radiation on MMP14 mRNA expression and increased TIMP1 expression in radiated cells (Figure 8B).

Of other angiogenic factors involved in angiogenesis, radiation increased CXCL6, CCL2, ERK1, ERK2, and AKT1 mRNA expression and decreased NOS3 mRNA expression. Melatonin counteracted the stimulatory effect of radiation on CXCL6, CCL2, ERK1, ERK2, and AKT1 mRNA expression. Melatonin increased significantly the inhibitory effect of ionizing radiation on NOS3 expression (Figure 8C).

### Effects of Melatonin on Changes Induced by Radiation on AKT and ERK Signal Pathways

**FIGURE 9:**
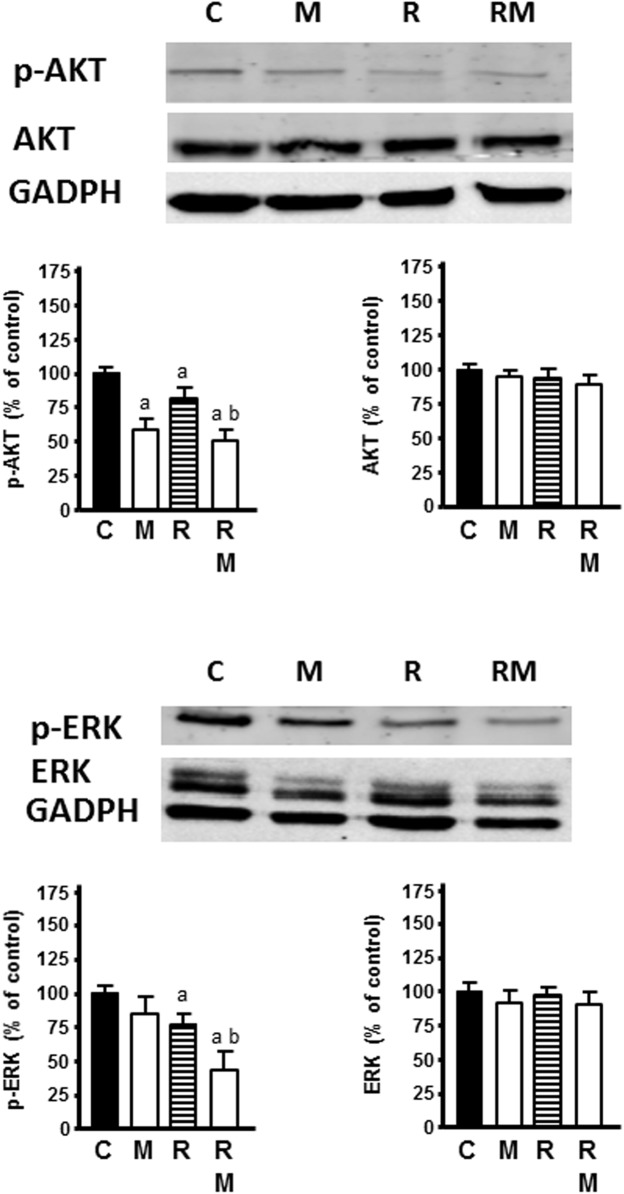
Effects of radiation in the presence or not of melatonin 1 mM on AKT and ERK signaling pathways. Western blot showing protein levels for AKT, ERK, p-AKT, p-ERK, and GADPH as a control. Data are expressed as the percentage of the control group (Mean ± SEM). C, control; M, melatonin; and R, radiation. a, *p* < 0.05 *vs* C; and b, *p* < 0.05 *vs* R.

Radiation markedly inhibited the activation of p-AKT and p-ERK, whereas no change of total AKT or ERK protein expression level was found. Melatonin pretreatment (1 mM) significantly increased the inhibitory effect of radiation on p-AKT and p-ERK (Figure 9).

### Effects of Melatonin on Ionizing Radiation-Induced Changes on Endothelial Cell Permeability

**FIGURE 10:**
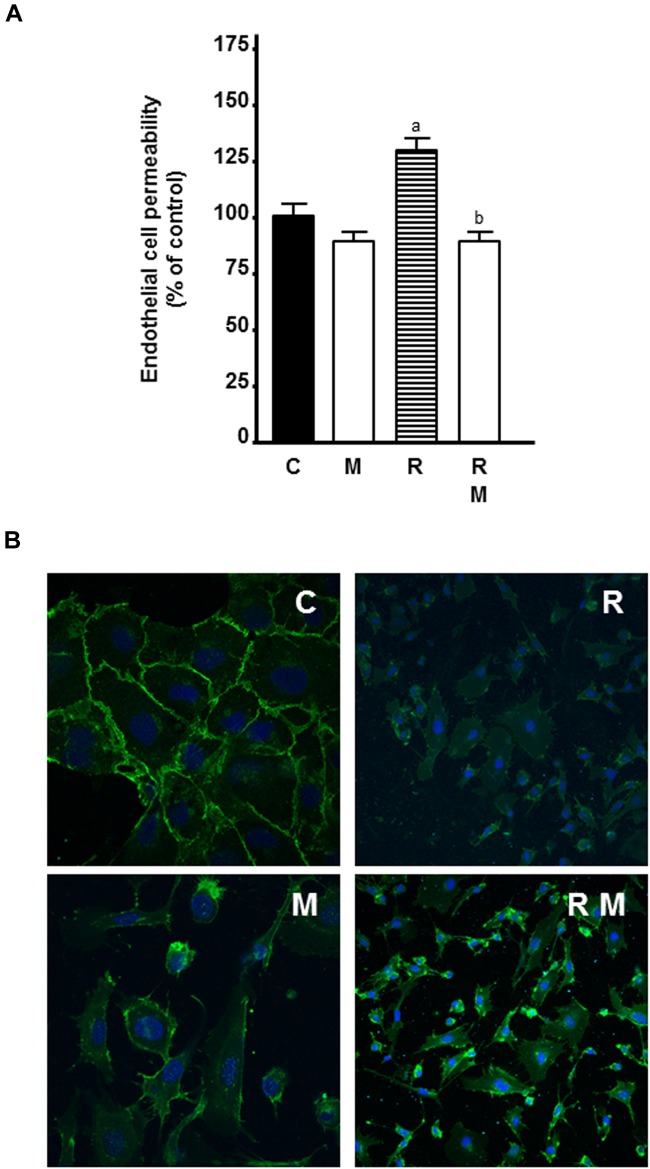
Effects of melatonin on radiation-induced changes on endothelial cell permeability and VE-cadherin internalization. **(A)** HUVECs were seeded onto 0, 1% collagen-coated 24-well insert wells until they reached full confluence. Then, VEGF 4 ng/ml was added to the plates and inserts and they were radiated. After 24 h, FITC-dextran solution was added and its passage into the lower chamber was measured at 90 min. Data are expressed as the percentage of the control group (Mean ± SEM). a, *p* < 0.05 *vs* C; and b, *p* < 0.01 *vs* R. **(B)** HUVECs grown on coverslips were irradiated and, after 24 h, processed for immunofluorescence studies of the internalization of VE-cadherin as described in material and methods. C, control; M, melatonin; and R, radiation.

To study whether the use of melatonin as adjuvant to radiotherapy could also have an effect on vessel normalization, we investigated the effects of melatonin pretreatment on the permeability of HUVECs in radiated cells. As showed in Figure 10, 8 Gy radiation alone increased endothelial cell permeability. Melatonin counteracted significantly the stimulatory effect of radiation.

Since melatonin decreased endothelial cell permeability and VE-cadherin is the major tight junction proteins needed to maintain the endothelial cell barrier, we studied the effect of melatonin on VE-cadherin distribution by immunostaining. Control cells showed continuous VE-cadherin staining in cell to cell contact. Radiation disrupted this pattern and led to intracellular accumulation of VE-cadherin with shortening of the width of VE-cadherin-positive areas. Melatonin prevented radiation-mediated VE-cadherin internalization from the cell surface and increased VE-cadherin staining at cell-cell junctions (Figure 10).

### Influence of Melatonin on Radiation-Induced Effects on New Formed Blood Vessels in an *in vivo* Angiogenesis Assay

**FIGURE 11:**
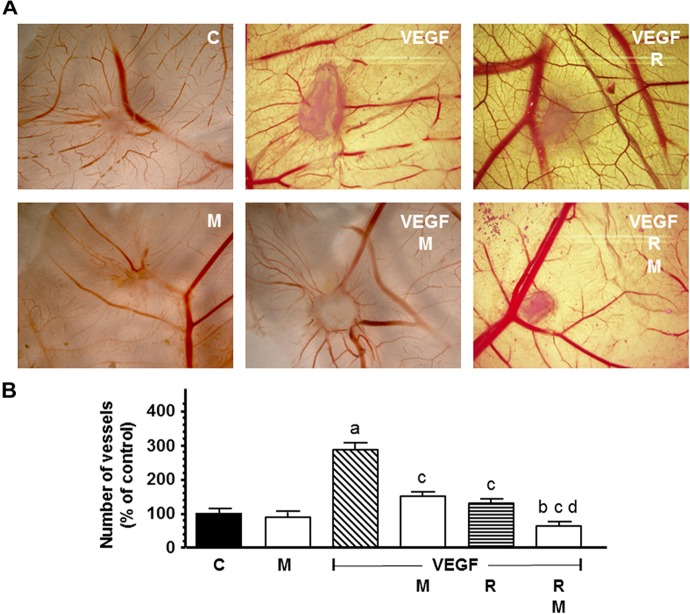
*In vivo* effect of melatonin on the radiation-induced actions on the VEGF-stimulated angiogenesis in the chorioallantoic membrane assay. Eggs were irradiated and alginate beads with VEGF 1.57 pM, as stimulator of blood vessel formation, with and without melatonin 1 mM, were placed on the chorioallantoic membrane on day 11 of development. On day 14, newly formed blood vessel converging toward the alginates were counted at microscopic level. **(A)** Representative images of CAM. **(B)** Vessel number data shown are expressed as mean ± SEM. C, control; M, melatonin; VEGF, vascular endothelial growth factor; and R, radiation. a, *p* < 0.001 vs C; b, *p* < 0.05 vs C; c, *p* < 0.001 vs VEGF; and d, *p* < 0.001 vs R.

The new formed blood vessels branch points were studied in the chick CAM assay, an *in vivo* angiogenesis assay. As showed in Figure 11, VEGF alone showed a potent angiogenic response. Radiation significantly decreased the VEGF-induced angiogenesis. Melatonin increased significantly (*p* < 0.001) the reduction of the vascular area induced by radiation.

## Discussion

Radiotherapy is a treatment option in more than half of patients with malignant tumors. One important objective in radiobiology is to increase the sensitivity of malignant cells to the radiotherapy and, at the same time, to preserve normal cells (Allison and Dicker, 2014). The use of radiosensitizers can decrease side effects in healthy tissues and improve cancer cell response to radiotherapy. Some of the radiosensitizers that has been combined with the radiation are antiangiogenic agents. Since tumor vascular neo-angiogenesis has an outstanding role in tumor ontogenesis and progression, the inhibition of tumoral angiogenesis has also become an anticancer strategy in line with other treatments, such as radiotherapy, chemotherapy and surgery (Wang et al., 2015). Melatonin has been described as an inhibitor of neoplastic growth through different mechanisms that can increase the therapeutic effects of ionizing radiation as well as chemotherapy (Martínez-Campa et al., 2017; Najafi et al., 2017; Farhood et al., 2019). Among the mechanisms of the antitumor action of melatonin are antiangiogenic effects, which has proven useful in breast cancer treatment in combination with some chemotherapeutic drugs (Alvarez-García et al., 2013b,c; Martínez-Campa et al., 2017; Alonso-González et al., 2018). Therefore, in the current study, we investigated the effects of ionizing radiation on different steps of the angiogenic process and studied the modulation of these actions by melatonin at endothelial cell level.

The present study demonstrates that melatonin potentiates antiangiogenic actions and neutralizes proangiogenic actions induced by ionizing radiation. We used different assays to study different aspects of angiogenic behavior. The first step of angiogenesis is the proliferation of endothelial cells. Radiation, at 4, 6, 8, or 10 Gy, decreased endothelial cell proliferation, as it has been previously reported, without large differences between the different doses of radiation, probably because the incubation time of 3 days was short (Kargiotis et al., 2010; Kim et al., 2015). Melatonin at 1 mM concentration alone, according with previous findings, induced an inhibitory effect on the proliferation of endothelial cells (Alvarez-García et al., 2013c). We used this melatonin concentration because was the most effective doses in endothelial cells in previous works (Alvarez-García et al., 2013b,c). In addition, melatonin pretreatment potentiated the inhibitory effect on cell proliferation induced by ionizing radiation. This potentiation of the antiproliferative effects of radiation on endothelial cell agrees with recent findings in which melatonin enhanced the antiproliferative effect induced by ionizing radiation, on human breast cancer cells, and modulated changes induced by radiation in gene expression involved in double-strand DNA break repair and estrogen biosynthesis (Alonso-González et al., 2015, 2016, 2018). We then analyzed the changes induced by ionizing radiation in other steps of the angiogenesis process like endothelial cells migration or the tubular network formation. Cell irradiation have been reported that inhibits HUVECs migration and the three-dimensional organization of the endothelial cells into capillary-like tubes (Kargiotis et al., 2010; Fokas et al., 2012; Kaffas et al., 2014; Kim et al., 2015). In this study, we found that ionizing radiation did not significantly modified the migration of endothelial cells but, effectively, decreased the tubular network formation. The different effects of ionizing radiation on the tumor vasculature can be explained by their dependence on the dose and scheduling of radiation. High doses, above 10 Gy, induces vascular damage that ends in cell death. However, fractionated low dose radiation, like daily fractions of up to 2 Gy induces a positive effect on tumor vasculature (Goedegebuure et al., 2019). Melatonin reduced endothelial cell migration and significantly enhanced the reduction of tubule area induced by radiation. Our results are in agreement with those that describe antiangiogenic effects of melatonin in endothelial cell cultures. Melatonin inhibits cell migration, disrupts tube formation, counteracts the VEGF-stimulated tubular network formation by HUVECs and disintegrates preformed capillary network (Alvarez-García et al., 2013a). Other antiangiogenic compounds have obtained similar effects that melatonin, thus, the combination of ionizing radiation with sunitinib, an inhibitor of angiogenesis, enhanced the radiation response of endothelial cells and completely inhibited tube formation (Kaffas et al., 2014). The anti-angiogenic compound SU5416 has shown radiosensitizing effects in endothelial cells. These effects have been associated with inhibition of cell survival, inhibition of DNA repair activity, stimulation of apoptosis, inhibition of cell migration and invasion and suppression of angiogenesis (Kim et al., 2015).

Estrogens stimulate endothelial cell proliferation *in vitro* and *in vivo*, enhance adhesion of endothelial cells to various matrix proteins and increase cell migration, thus promoting angiogenesis (Trenti et al., 2018). The mechanisms responsible for the proangiogenic effect of estrogens have been widely investigated and appear to be largely mediated by ERα activation. In breast cancer, a clear association has been described between estrogen, ER expression by endothelial cells, angiogenic activity and/or tumor invasiveness (Trenti et al., 2018). For this reason, we considered interesting to study the effects of ionizing radiation and its combination with melatonin on activity and expression of aromatase, sulfatase and 17β-HSD1, enzymes involved in estrogen biosynthesis in endothelial cells. In co-cultures of endothelial and mammary tumor cells, radiation downregulated aromatase mRNA expression, the aromatase endothelial-specific promoter I.7, sulfatase activity and expression and 17β-HSD1 activity and expression. Melatonin potentiated the effect induced by ionizing radiation. At this moment, it is not described the effect of ionizing radiation on the estrogen biosynthesis in endothelial cells. It has been reported only that chemotherapeutic agents like docetaxel decrease intratumoral aromatase mRNA levels in breast tumors, suggesting that antitumor activity of chemotherapy is mediated, at least in part, through suppression of intratumoral estrogens synthesis (Miyoshi et al., 2004). However, it is well known that melatonin regulates the synthesis and transformation of androgens and active estrogens in human breast cancer cells and endothelial cells, through the inhibition of aromatase, sulfatase and 17β-HSD1 activity and expression (Cos et al., 2008; Alvarez-García et al., 2013b; Alonso-González et al., 2016).

We also studied melatonin effects on some of the main pro-angiogenic factors. VEGF is the most active endogenous pro-angiogenic factor, which induces angiogenesis directly on the endothelial cells (Liang and Hyder, 2005). More recently, the ANG/Tie2 receptor signaling are considered together with VEGF the main regulators of different mechanisms of tumor vascularization that act in a complementary and coordinated way (Thomas and Augustin, 2009; Danza et al., 2013). Both are ligands of the Tie2 tyrosine kinase receptor. ANG-1 binds to Tie2 receptor and induces vessel maturation and stabilizes tumor vasculature. However, ANG-2 competes with ANG-1 for Tie2 binding, causing vessel regression in the absence of VEGF, whereas it promotes angiogenesis in the presence of VEGF (Holash et al., 1999; Fagiani and Christofori, 2013). Radiation and melatonin induced a significant decrease in ANG-1, ANG-2, and VEGF mRNA expression. Melatonin pretreatment before irradiation potentiated the inhibitory effect induced by radiation on ANG-2 and VEGF expression. It is known that high dose of radiation inhibits angiogenesis by inhibiting endothelial cell survival, proliferation, migration and tube formation. However, low dose of radiation can also induce VEGF production and protect tumor blood vessels, resulting in tumor radioresistance (Gorski et al., 1999; Kobayashi and Lin, 2006). Depriving endothelial cells of VEGF may represent an important strategy to increase the antitumor effects of ionizing radiation and many studies describe that irradiation after antiangiogenic therapy sensitize endothelial cells and enhances the effect of radiotherapy (Gorski et al., 1999; Kobayashi and Lin, 2006). The overexpression of VEGF has been related with an increase of endothelial cell survival and tumor radioresistance (Gorski et al., 1999; Gupta et al., 2002). Wachsberger et al. (2003) proposed that enhancement of radiation response occurs through inhibition of VEGF-induced protection against radiation damage to endothelial cells. It is known that melatonin decreases the production and the expression of VEGF in human breast cancer cells and coordinates at the same time a downregulation of angiopoietins with a reduction of VEGF in cocultures of endothelial and breast cancer cells (Alvarez-García et al., 2013b,c; González et al., 2017). Melatonin has antiangiogenic actions by downregulation of VEGF in several kind of tumors, such as neuroblastoma (González et al., 2017), breast cancer (Alvarez-García et al., 2013b,c), ovarian (Zonta et al., 2017), gastric (Wang et al., 2016), pancreatic (Cui et al., 2012), prostatic (Sohn et al., 2015), or hepatocarcinoma (Colombo et al., 2016). In addition, in Walker 256 carcinoma and mandibular salivary gland of rats during paraneoplastic process, melatonin in combination with cyclophosphamide reduced tumor growth and the expression of VEGF-A in tumor and salivary gland, a marker in early diagnostics of neoplasms and estimation of the efficiency of antitumor therapy (Ovsyanko et al., 2015). The potentiation by melatonin of the inhibitory effect induced by radiation on VEGF expression could be an important mechanism through which melatonin increases tumor radiosensitivity.

When we examined the changes in the expression of 84 genes related to angiogenesis in endothelial cells irradiated in the presence or not of melatonin, we found that, in relation with the regulation of angiogenic factors, radiation increased JAG1, IGF-1, KDR, FGFR3, and TGF_α_ expression. JAG1 has been implicated in different aspects of cancer biology including tumor angiogenesis, neoplastic cell growth and the metastatic process (Grochowski et al., 2016). JAG1 mRNA expression is upregulated in breast cancer and has been correlated with a poor overall breast cancer survival (Reedijk et al., 2005; Grochowski et al., 2016). IGF-1 and KDR are involved in promoting tumoral cell growth, neoplastic transformation processes, angiogenesis, cell migration and tumor progression (Oh et al., 2002; Liang and Hyder, 2005). FGFR3 expression is associated with poor prognosis in breast cancer (Wang and Ding, 2017). In addition, recent studies also indicate that FGFR3 may function via a common molecular pathway causing breast cancer and may be a candidate therapeutic target in FGFR3-associated breast cancer (Wang and Ding, 2017). TGF_α_ acts synergistically with TGF_β_ promoting cell proliferation (Gacche and Meshram, 2013). In our study melatonin counteracted the stimulatory effect of radiation on the five angiogenic factors. It should be noted that the stimulatory effects of radiation on these angiogenic factors expression could be considered as a negative effect because they are increasing angiogenesis. However, melatonin counteracted this negative effect and neutralized this action.

In regard to the modulation of extracellular matrix molecules involved in angiogenesis, radiation increased MMP14 expression. MMP14 is a metalloproteinase which has been found to play a critical role in cancer invasion and metastasis by cleaving extracellular matrix and basement membrane proteins, activating proMMP-2 and -13, inducing the activation of growth factors, and enhancing cell migration (Seiki, 2003). In our study, melatonin counteracted the stimulatory effect of radiation on MMP14 expression and increased TIMP1 expression. TIMP1 influences processes as survival, migration or proliferation through indirect mechanisms based on the inhibition of metalloproteinases and its stimulation can prevent angiogenesis *in vivo* (Zacchigna et al., 2004; Ries, 2014). Our results are in accordance with those of Ordoñez et al. (2014) which described that melatonin regulates motility and invasiveness of human HepG2 hepatocarcinoma cells, through a mechanism that involves TIMP-1 upregulation and attenuation of MMP-9 expression and activity via NF-jB signal pathway inhibition.

Finally, in relation with other angiogenic factors involved in angiogenesis radiation increased CXCL6, CCL2, ERK1, ERK2, and AKT1 expression and decreased NOS3 expression. CXCL6 mediates breast cancer progression by pERK1/2-dependent mechanisms and stimulates endothelial cell proliferation and migration (Ma et al., 2017). CCL2 induces the invasive phenotype of human breast epithelial cells (Lee et al., 2018). It has also been described a correlation between NOS3 expression and progression of malignancy in human breast cancer (Vannini et al., 2015). ERK1, ERK2, and the protein kinase AKT1 are mediators for several signal transduction pathways through its ability to phosphorylate different downstream targets directly involved in cellular processes related with vascular remodeling, including proliferation, cell survival, and metabolism (Yu et al., 2015). Melatonin counteracted the stimulatory effect of radiation on CXCL6, CCL2, ERK1, ERK2, and AKT1 expression and increased significantly the inhibitory effect of ionizing radiation on NOS3 expression. This result agree with previous findings, in hepatocellular carcinoma cell lines, where melatonin also downregulated CXCL6 at pharmacologic concentrations (Lin and Chuang, 2010). In human endothelial cells, intracellular signaling pathways, such as MAPK, PI3K/ AKT, ERK1/2 pathways, involved in VE cell proliferation, survival and migration, are inactivated by melatonin which finally lead to the decrease of endothelial cell proliferation (Cui et al., 2008). Hypoxia-induced migration of endothelial cells is inhibited through the inhibition of ERK/Rac1 pathway (Yang et al., 2014). Recently, regulation by melatonin of main signal transduction pathways, such as AKT and MAPK, has been also related with reduction of drug resistance in cancer chemotherapy (Asghari et al., 2018). Another melatonin inhibitory mechanism in tumoral angiogenesis has also been previously related with the inhibition of sphingosine kinase 1 which affect the stability of HIF-1α through AKT-GSK-3β pathway (Cho et al., 2011). In our study, melatonin counteracted the increase of AKT1 and ERK 1/2 expression and potentiated the inhibitory effect on the activation of p-AKT and p-ERK exerted by radiation. These results suggest that the antiproliferative and antiangiogenic effect of melatonin could be mediated through AKT and ERK pathways, two of the intracellular signaling pathways involved in many important physiological processes, such as VE cell growth, cell proliferation, survival, migration and angiogenesis.

Since permeability is a main determinant of vascular function we studied if radiation and melatonin could modulate it. Radiation increased endothelial cell permeability and melatonin counteracted the stimulatory effect of radiation. Recently, it has been described that radiation enhances endothelial cell permeability through changes in VE-cadherin (Kishimoto et al., 2018). VE-cadherin is one of the most important junction proteins of the endothelial cells that regulates vascular permeability (Rho et al., 2017) and is accumulated in cell to cell contact. However, radiation induced an intracellular accumulation of VE-cadherin. Melatonin counteracted the effects of radiation and decreased the levels of intracellular VE-cadherin. It is known that phosphorylation and internalization of VE-cadherin led to an increase of endothelial cell permeability, which is involved in tumor angiogenesis (Rho et al., 2017). Phosphorylation and internalization of VE-cadherin is induced by permeability-increasing agents, such as VEGF (Gavard, 2014). Again, the inhibition of VEGF by melatonin could explain the inhibition of cell permeability exerted by melatonin. In addition, the MAPK/ERK and PI3K/AKT pathways are also involved in VEGF-induced permeability in HUVECs (Jiang and Liu, 2008). Thus, melatonin could inhibit endothelial cell permeability by regulating the internalization of VE-cadherin, through the inhibition of VEGF and by reducing the phosphorylation of AKT and ERK.

In the CAM assay, an *in vivo* model of neovessel formation, melatonin increased the reduction of the vascular area induced by radiation. To our knowledge this is the first time that the inhibition of angiogenesis by melatonin in combination with ionizing radiation is described in this *in vivo* model.

Radiation kills cancer cells but, sometimes, induces a more aggressive and malignant postradiation tumor, due to the development of resistant cancer cells with increased proliferative, invasive and angiogenic properties. In order to improve its effectiveness there is growing interest in combining radiotherapy with other therapies, such as antiangiogenic or immune therapies (Goedegebuure et al., 2019). The effects of ionizing radiation on angiogenesis depend on the balance between angiogenic promoters and inhibitors and the expression of the different genes involved in this process must be coordinated both quantitatively and temporally to ensure correct angiogenesis. In our study, melatonin treatment counteracted some of these negative actions of radiation and coordinated better the balance between stimulating factors and inhibitors of angiogenesis.

In summary, the results of our study indicate that melatonin enhances the inhibitory effects on angiogenesis induced by radiation in human endothelial cells. Melatonin potentiated antiangiogenic actions and enhanced radiation-induced inhibitory effect on several steps of the angiogenic process (cell proliferation, tubular network formation), on the enzymes involved in estrogen biosynthesis (aromatase mRNA expression, aromatase promoter I.7, sulfatase mRNA expression and 17β-HSD1 activity and expression), on some proangiogenic factors, such as ANG-2 and VEGF. In addition, melatonin neutralized the stimulatory effect of radiation on endothelial cell permeability and on the expression of some proangiogenic genes, like FGFR3, TGF_α_, IGF-1, KDR, JAG1, MMP14, CXCL6, CCL2, ERK1, ERK2, and AKT1. *In vivo*, melatonin also counteracted the stimulatory effect of VEGF on angiogenesis and enhanced the inhibitory effect of radiation. Antiangiogenic effect of melatonin could be mediated through AKT and ERK pathways since melatonin potentiated the inhibitory effect on the activation of p-AKT and p-ERK exerted by radiation. Furthermore, melatonin can also inhibit VEGF-induced angiogenesis by reducing cell permeability and influencing the internalization of VE-cadherin. One of the most valuable part of this work have been to demonstrate that radiation has some side effects on angiogenesis that may reduce its effectiveness against tumor growth and that melatonin is able to neutralize these negative actions of ionizing radiation. Our study has some limitations, as we measured mRNA expression of several proteins involved in angiogenesis but we did not measured protein content in all of them. This melatonin radiosensitive effect may allow to reduce the dose of radiation, thus enhancing its antitumor action. Our findings point to melatonin as a useful molecule as adjuvant to radiotherapy in cancer treatment.

## Data Availability

The datasets for this manuscript are not publicly available because all relevant data are included in the manuscript. Requests to access the datasets should be directed to gonzalav@unican.es.

## Author Contributions

AG-G carried out the culture experiments, measurement of cellular proliferation, enzymatic activities, CAM assay, cell migration, cell differentiation assays, and RT-PCR. CM-C and AG were involved in the conception and design of the study, and data analysis and interpretation. NR performed the Western blot experiments. CA-G and JM-M were involved in the analysis of the angiogenesis array. JG-A was involved in cell irradiation. SC conceived the experiments, and was involved in the study design, data analysis, and manuscript writing. All authors reviewed the manuscript and gave final approval to the submitted version.

## Conflict of Interest Statement

The authors declare that the research was conducted in the absence of any commercial or financial relationships that could be construed as a potential conflict of interest.

## References

[B1] Ackerman G. E. Smith M. E. Mendelson C. R. MacDonald P. C. Simpson E. R. (1981). Aromatization of androstenedione by human adipose tissue stromal cells in monolayer culture. *J. Clin. Endocrinol. Metab.* 53 412–417. 10.1210/jcem-53-2-412 7251819

[B2] Allison R. Dicker A. (2014). Minimizing morbidity in radiation oncology: a special issue from future oncology. *Future Oncol.* 10 2303–2305. 10.2217/fon.14.195 25525839

[B3] Alonso-González C. González A. Martínez-Campa C. Gómez-Arozamena J. Cos S. (2015). Melatonin sensitizes human breast cancer cells to ionizing radiation by downregulating proteins involved in double-strand DNA break repair. *J. Pineal Res.* 58 189–197. 10.1111/jpi.12205 25623566

[B4] Alonso-González C. González A. Martínez-Campa C. Menéndez-Menéndez J. Gómez-Arozamena J. García-Vidal A. (2016). Melatonin enhancement of the radiosensitivity of human breast cancer cells is associated with the modulation of proteins involved in estrogen biosynthesis. *Cancer Lett.* 370 145–152. 10.1016/j.canlet.2015.10.015 26497762

[B5] Alonso-González C. Menéndez-Menéndez J. González-González A. González A. Cos S. Martínez-Campa C. (2018). Melatonin enhances the apoptotic effects and modulates the changes in gene expression induced by docetaxel in MCF-7 human breast cancer cells. *Int. J. Oncol.* 52 560–570. 10.3892/ijo.2017.4213 29207126

[B6] Alvarez-García V. González A. Alonso-González C. Martínez-Campa C. Cos S. (2012). Melatonin interferes in the desmoplastic reaction in breast cancer by regulating cytokine production. *J. Pineal Res.* 52 282–290. 10.1111/j.1600-079X.2011.00940.x 22151118

[B7] Alvarez-García V. González A. Alonso-González C. Martínez-Campa C. Cos S. (2013a). Antiangiogenic effects of melatonin in endothelial cell cultures. *Microvasc. Res.* 87 25–33. 10.1016/j.mvr.2013.02.008 23473980

[B8] Alvarez-García V. González A. Alonso-González C. Martínez-Campa C. Cos S. (2013b). Regulation of vascular endothelial growth factor by melatonin in human breast cancer cells. *J. Pineal Res.* 54 373–380. 23013414 10.1111/jpi.12007

[B9] Alvarez-García V. González A. Martínez-Campa C. Alonso-González C. Cos S. (2013c). Melatonin modulates aromatase activity and expression in endothelial cells. *Oncol. Rep.* 29 2058–2064. 10.3892/or.2013.2314 23450505

[B10] Asghari M. H. Ghobadi E. Moloudizargari M. Fallah M. Abdollahi M. (2018). Does the use of melatonin overcome drug resistance in cancer chemotherapy? *Life Sci.* 196 143–155. 10.1016/j.lfs.2018.01.024 29374563

[B11] Bagheri H. Rezapour S. Najafi M. Motevaseli E. Shekarchi B. Cheki M. (2018). Protection against radiation-induced micronuclei in rat bone marrow erythrocytes by curcumin and selenium L-methionine. *Iran J. Med. Sci.* 43 645–652. 30510341 PMC6230935

[B12] Bareschino M. A. Schettino C. Colantuoni G. Rossi E. Rossi A. Maione P. (2011). The role of antiangiogenic agents in the treatment of breast cancer. *Curr. Med. Chem.* 18 5022–5032. 22050750 10.2174/092986711797636072

[B13] Bergers G. Benjamin L. E. (2003). Tumorigenesis and the antiangiogenic switch. *Nat. Rev. Cancer* 3 401–410. 10.1038/nrc1093 12778130

[B14] Bhadada S. V. Goyal B. R. Patel M. M. (2011). Angiogenic targets for potential disorders. *Fund. Clin. Pharmacol.* 25 29–47. 10.1111/j.1472-8206.2010.00814.x 20199582

[B15] Blask D. E. Dauchy R. T. Sauer L. A. Krause J. A. Brainard G. C. (2003). Growth and fatty acid metabolism of human breast cancer (MCF-7) xenografts in nude rats: impact of constant light-induced nocturnal melatonin suppression. *Breast Cancer Res. Treat.* 79 313–320. 10.1023/a:1024030518065 12846415

[B16] Cho S. Y. Lee H. J. Jeong S. J. Lee H. J. Kim H. S. Chen C. Y. (2011). Sphingosine kinase 1 pathway is involved in melatonin-induced HIF-1α in activation in hypoxic PC-3 prostate cancer cells. *J. Pineal Res.* 51 87–93. 10.1111/j.1600-079X.2011.00865.x 21392092

[B17] Colombo J. Maciel J. M. Ferreira L. C. Da Silva R. F. Zuccari D. A. (2016). Effects of melatonin on HIF-1α and VEGF expression and on the invasive properties of hepatocarcinoma cells. *Oncol. Lett.* 12 231–237. 10.3892/ol.2016.4605 27347130 PMC4907066

[B18] Cook K. M. Figg W. D. (2010). Angiogenesis inhibitors: current strategies and future prospects. *CA Cancer J. Clin.* 60 222–243. 10.3322/caac.20075 20554717 PMC2919227

[B19] Cos S. Álvarez-García V. González A. Alonso-González C. Martínez-Campa C. (2014). Melatonin modulation of crosstalk among malignant epithelial, endothelial and adipose cells in breast cancer. *Oncol. Lett.* 8 487–492. 10.3892/ol.2014.2203 25009641 PMC4081418

[B20] Cos S. Blask D. E. Lemus-Wilson A. Hill A. B. (1991). Effects of melatonin on the cell cycle kinetics and estrogen rescue of MCF-7 human breast cancer cells in culture. *J. Pineal Res.* 10 36–42. 10.1111/j.1600-079x.1991.tb00007.x 2056430

[B21] Cos S. González A. Martínez-Campa C. Mediavilla M. D. Alonso-González C. Sánchez-Barceló E. J. (2008). Melatonin as a selective estrogen enzyme modulator. *Curr. Cancer Drug Tar.* 8 691–702. 10.2174/156800908786733469 19075592

[B22] Cos S. Sánchez-Barceló E. J. (2000). Melatonin and mammary pathological growth. *Front. Neuroendocrinol.* 21 133–170. 10.1006/frne.1999.0194 10764528

[B23] Cui P. Yu M. Luo Z. Dai M. Han J. Xiu R. (2008). Intracellular signaling pathways involved in cell growth inhibition of human umbilical vein endothelial cells by melatonin. *J. Pineal Res.* 44 107–114. 18078456 10.1111/j.1600-079X.2007.00496.x

[B24] Cui P. Yu M. Peng X. Dong L. Yang Z. (2012). Melatonin prevents human pancreatic carcinoma cell PANC-1-induced human umbilical vein endothelial cell proliferation and migration by inhibiting vascular endothelial growth factor expression. *J. Pineal Res.* 52 236–243. 10.1111/j.1600-079X.2011.00933.x 21913973

[B25] Danza K. Pilato B. Lacalamita R. Addati T. Giotta F. Bruno A. (2013). Angiogenetic axis angiopoietins/Tie2 and VEGF in familial breast cancer. *Eur. J. Hum. Genet.* 21 824–830. 10.1038/ejhg.2012.273 23232696 PMC3722683

[B26] Duncan L. Purohit A. Howarth N. M. Potter B. V. Reed M. J. (1993). Inhibition of estrone sulfatase activity by estrone-3-methylthiophosphonate: a potential therapeutic agent in breast cancer. *Cancer Res.* 53 298–303. 8417823

[B27] Dvorak H. F. Weaver V. M. Tlsty T. D. Bergers G. (2011). Tumor microenvironment and progression. *J. Surg. Oncol.* 103 468–474. 10.1002/jso.21709 21480238 PMC3277953

[B28] Fagiani E. Christofori G. (2013). Angiopoietins in angiogenesis. *Cancer Lett.* 328 18–26. 10.1016/j.canlet.2012.08.018 22922303

[B29] Fandrey J. Genius J. (2000). Reactive oxygen species as regulators of oxygen dependent gene expression. *Adv. Exp. Med. Biol.* 475 153–159. 10.1007/0-306-46825-5_15 10849657

[B30] Farhood B. Goradel N. H. Mortezaee K. Khanlarkhani N. Salehi E. Nashtaei M. S. (2019). Melatonin as an adjuvant in radiotherapy for radioprotection and radiosensitization. *Clin. Transl. Oncol.* 21 268–279. 10.1007/s12094-018-1934-0 30136132

[B31] Fokas E. Yoshimura M. Prevo R. Higgins G. Hackl W. Maira S. M. (2012). NVP-BEZ235 and NVP-BGT226, dual phosphatidylinositol 3-kinase/mammalian target of rapamycin inhibitors, enhance tumor and endothelial cell radiosensitivity. *Radiat. Oncol.* 7:48. 10.1186/1748-717X-7-48 22452803 PMC3348043

[B32] Gacche R. N. Meshram R. J. (2013). Targeting tumor micro-environment for design and development of novel anti-angiogenic agents arresting tumor growth. *Prog. Biophys. Mol. Biol.* 113 333–354. 10.1016/j.pbiomolbio.2013.10.001 24139944

[B33] Gavard J. (2014). Endothelial permeability and VE-cadherin: a wacky comradeship. *Cell Adh. Migr.* 8 158–164. 10.4161/cam.29026 25422846 PMC4049861

[B34] Goedegebuure R. S. A. Klerk L. K. Bass A. J. Derks S. Thijssen V. L. J. L. (2019). Combining radiotherapy with anti-angiogenic therapy and immunotherapy; a therapeutic triad for cancer? *Front. Immunol.* 9:3107. 10.3389/fimmu.2018.03107 30692993 PMC6339950

[B35] González A. Alvarez-García V. Martínez-Campa C. M. Alonso-González C. Cos S. (2012). Melatonin promotes differentiation of 3T3-L1 fibroblasts. *J. Pineal Res.* 52 12–20. 10.1111/j.1600-079X.2011.00911.x 21718362

[B36] González A. González-González A. Alonso-González C. Menéndez-Menéndez J. Martínez-Campa C. Cos S. (2017). Melatonin inhibits angiogenesis in SH-SY5Y human neuroblastoma cells by downregulation of VEGF. *Oncol. Rep.* 37 2433–2440. 10.3892/or.2017.5446 28259965

[B37] González A. Martínez-Campa C. Mediavilla M. D. Alonso-González C. Sánchez-Barceló E. J. Cos S. (2007). Inhibitory effects of pharmacological doses of melatonin on aromatase activity and expression in rat glioma cells. *Br. J. Cancer* 97 755–760. 10.1038/sj.bjc.6603935 17700567 PMC2360391

[B38] González-González A. González A. Alonzo-González C. Menéndez-Menéndez J. Martínez-Campa C. Cos S. (2018). Complementary actions of melatonin on angiogenic factors (angiopoietins/Tie2 axis and VEGF) in co-cultures of human endothelial and breast cancer cells. *Oncol. Report.* 39 433–441. 10.3892/or.2017.6070 29115538

[B39] Gorski D. H. Beckett M. A. Jaskowiak N. T. Calvin D. P. Mauceri H. J. Salloum R. M. (1999). Blockade of the vascular endothelial growth factor stress response increases the antitumor effects of ionizing radiation. *Cancer Res.* 59 3374–3378. 10416597

[B40] Grochowski C. M. Loomes K. M. Spinner N. B. (2016). Jagged1 (JAG1): structure, expression, and disease associations. *Gene* 576 381–384. 10.1016/j.gene.2015.10.065 26548814 PMC4673022

[B41] Gupta V. K. Jaskowiak N. T. Beckett M. A. Mauceri H. J. Grunstein J. Johnson R. S. (2002). Vascular endothelial growth factor enhances endothelial cell survival and tumour radioresistance. *Cancer J.* 8 47–54. 10.1097/00130404-200201000-00009 11895203

[B42] Hill S. M. Blask D. E. (1998). Effects of the pineal hormone melatonin on the proliferation and morphological characteristics of human breast cancer cells (MCF-7) in culture. *Cancer Res.* 48 6121–6126. 3167858

[B43] Holash J. Wiegand S. J. Yancopoulos G. D. (1999). New model of tumor angiogenesis: dynamic balance between vessel regression and growth mediated by angiopoietins and VEGF. *Oncogene* 18 5356–5362. 10.1038/sj.onc.1203035 10498889

[B44] Jiang B. H. Liu L. Z. (2008). AKT signaling in regulating angiogenesis. *Curr. Cancer Drug Targets* 8 19–26. 10.2174/156800908783497122 18288940

[B45] Kaffas A. E. Al-Mahrouki A. Tran W. T. Giles A. Czarnota G. J. (2014). Sunitinib effects on the radiation response of endothelial and breast tumor cells. *Microvasc. Res.* 92 1–9. 10.1016/j.mvr.2013.10.008 24215790

[B46] Kajdaniuk D. Marek B. Kos-Kudła B. Zwirska-Korczala K. Ostrowska Z. Buntner B. (2002). Does the negative correlation found in breast cancer patients between plasma melatonin and insulin-like growth factor-I concentrations imply the existence of an additional mechanism of oncostatic melatonin influence involved in defense? *Med. Sci. Monit*. 8 CR457–CR461. 12070440

[B47] Kargiotis O. Geka A. Rao J. S. Kyritsis A. P. (2010). Effects of irradiation on tumor cell survival, invasion and angiogenesis. *J. Neurooncol.* 100 323–338. 10.1007/s11060-010-0199-4 20449629

[B48] Kim E. H. Kim M. S. Jeong Y. K. Cho I. You S. H. Cho S. H. (2015). Mechanisms for SU5416 as a radiosensitizer of endothelial cells. *Int. J. Oncol.* 47 1440–1450. 10.3892/ijo.2015.3127 26314590

[B49] Kishimoto M. Akashi M. Kakei Y. Kusumoto J. Sakakibara A. Hasegawa T. (2018). Ionizing radiation enhances paracellular permeability through alteration of intercellular junctions in cultured human lymphatic endothelial cells. *Lymphat Res. Biol.* 16 390–396. 10.1089/lrb.2017.0072 29862914

[B50] Kobayashi H. Lin P. C. (2006). Antiangiogenic and radiotherapy for cancer treatment. *Histol. Histopathol.* 21 1125–1134. 10.14670/HH-21.1125 16835835

[B51] Lee S. Lee E. Ko E. Ham M. Lee H. M. Kim E. S. (2018). Tumor-associated macrophages secrete CCL2 and induce the invasive phenotype of human breast epithelial cells through upregulation of ERO1-α and MMP-9. *Cancer Lett.* 437 25–34. 10.1016/j.canlet.2018.08.025 30165193

[B52] Liang Y. Hyder S. M. (2005). Proliferation of endothelial and tumor epithelial cells by progestin-induced vascular endothelial growth factor from human breast cancer cells: paracrine and autocrine effects. *Endocrinology* 146 3632–3641. 10.1210/en.2005-0103 15845615

[B53] Lin Z. Y. Chuang W. L. (2010). Pharmacologic concentrations of melatonin have diverse influence on differential expressions of angiogenic chemokine genes in different hepatocellular carcinoma cell lines. *Biomed. Pharmacother.* 64 659–662. 10.1016/j.biopha.2010.09.006 20970952

[B54] Livak K. J. Schmittgen T. D. (2001). Analysis of relative gene expression data using real-time quantitative PCR and the 2(-ΔΔC(T)) method. *Methods* 25 402–408. 10.1006/meth.2001.1262 11846609

[B55] Ma J. C. Sun X. W. Su H. Chen Q. Guo T. K. Li Y. (2017). Fibroblast-derived CXCL12/SDF-1α promotes CXCL6 secretion and co-operatively enhances metastatic potential through the PI3K/Akt/mTOR pathway in colon cancer. *World J. Gastroenterol.* 23 5167–5178. 28811711 10.3748/wjg.v23.i28.5167 PMC5537183

[B56] Martínez-Campa C. Menéndez-Menéndez J. Alonso-González C. González A. Álvarez-García V. Cos S. (2017). What is known about melatonin, chemotherapy and altered gene expression in breast cancer. *Oncol. Rep.* 13 2003–2014. 10.3892/ol.2017.5712 28454355 PMC5403278

[B57] Mediavilla M. D. Sánchez-Barceló E. J. Tan D. X. Manchester L. Reiter R. J. (2010). Basic mechanisms involved in the anti-cancer effects of melatonin. *Curr. Med. Chem.* 17 4462–4481. 10.2174/092986710794183015 21062257

[B58] Meng L. I. Zhou J. Sasano H. Suzuki T. Zeitoun K. M. Bulun S. E. (2001). Tumor necrosis factor α and interleukin 11 secreted by malignant breast epithelial cells inhibit adipocyte differentiation by selectively down-regulating CCAAT/enhancer binding protein α and peroxisome proliferator-activated receptor γ: mechanism of desmoplastic reaction. *Cancer Res.* 61 2250–2255. 11280794

[B59] Miyoshi Y. Kim S. J. Akazawa K. Kamigaki S. Ueda S. Yanagisawa T. (2004). Down-regulation of intratumoral aromatase messenger RNA levels by docetaxel in human breast cancers. *Clin. Cancer Res.* 10 8163–8169. 10.1158/1078-0432.ccr-04-1310 15623590

[B60] Molis T. M. Spriggs L. L. Hill S. M. (1994). Modulation of estrogen receptor mRNA expression by melatonin in MCF-7 human breast cancer cells. *Mol. Endocrinol.* 8 1681–1690. 10.1210/mend.8.12.7708056 7708056

[B61] Mosmann T. (1983). Rapid colorimetric assay for cellular growth and survival: application to proliferation and cytotoxicity assays. *J. Immunol. Methods* 65 55–63. 10.1016/0022-1759(83)90303-4 6606682

[B62] Najafi M. Shirazi A. Motevaseli E. Geraily G. Norouzi F. Heidari M. (2017). The melatonin immunomodulatory actions in radiotherapy. *Biophys. Res.* 9 139–148. 10.1007/s12551-017-0256-8 28510090 PMC5425818

[B63] Narmani A. Farhood B. Haghi-Aminjan H. Mortezazadeh T. Aliasgharzadeh A. Mohseni M. (2018). Gadolinium nanoparticles as diagnosis and therapeutic agents: their delivery sistems in magnetic resonance imaging and neutron capture therapy. *J. Drug Deliv. Sci. Technol.* 44 457–466. 10.1016/j.jddst.2018.01.011

[B64] Oh J. S. Kucab J. E. Bushel P. R. Martin K. Bennett L. Collins J. (2002). Insulin-like growth factor-1 inscribes a gene expression profile for angiogenic factors and cancer progression in breast epithelial cells. *Neoplasia* 4 204–217. 10.1038/sj.neo.7900229 11988840 PMC1531694

[B65] Ordoñez R. Carbajo-Pescador S. Prieto-Dominguez N. García-Palomo A. González-Gallego J. Mauriz J. L. (2014). Inhibition of matrix metalloproteinase-9 and nuclear factor kappa B contribute to melatonin prevention of motility and invasiveness in HepG2 liver cancer cells. *J. Pineal Res.* 56 20–30. 10.1111/jpi.12092 24117795

[B66] Ovsyanko E. V. Kulishenko A. O. Lushnikova E. L. Vinogradov A. S. Zhurakovskii I. P. Pustovetova M. G. (2015). Expression of vascular endothelial growth factor (VEGF-A) in rat mandibular salivary gland during paraneoplastic process and treatment with cyclophosphamide and melatonin. *Bull. Exp. Biol. Med.* 158 676–680. 10.1007/s10517-015-2833-9 25778658

[B67] Porcù E. Salvador A. Primac I. Mitola S. Ronca R. Ravelli C. (2016). Vascular disrupting activity of combretastatin analogues. *Vascul. Pharmacol.* 83 78–89. 10.1016/j.vph.2016.05.006 27235861

[B68] Reedijk M. Odorcic S. Chang L. Zhang H. Miller N. McCready D. R. (2005). High level coexpression of JAG1 and NOTCH1 is observed in human breast cancer cell is associated with poor overall survival. *Cancer Res.* 65 8530–8537. 10.1158/0008-5472.can-05-1069 16166334

[B69] Reiter R. J. Rosales-Corral S. A. Tan D. X. (2017). Melatonin, a full service anti-cancer agent: inhibition of initiation, progression and metastasis. *Int. J. Mol. Sci.* 18:E843. 10.3390/ijms18040843 28420185 PMC5412427

[B70] Rho S. S. Ando K. Fukuhara S. (2017). Dynamic regulation of vascular permeability by vascular endothelial cadherin-mediated endothelial cell-cell junctions. *J. Nippon Med. Sch.* 84 148–159. 10.1272/jnms.84.148 28978894

[B71] Ries C. (2014). Cytokine functions of TIMP-1. *Cell Mol. Life Sci.* 71 659–672. 10.1007/s00018-013-1457-3 23982756 PMC11113289

[B72] Seiki M. (2003). Membrane-type I matrix metalloproteinase: a key enzyme for tumor invasion. *Cancer Lett.* 194 1–11. 10.1016/s0304-3835(02)00699-7 12706853

[B73] Sengupta S. Chattopadhyay M. K. (1993). Lowry’s method of protein estimation: some more insights. *J. Pharm. Pharmacol.* 45:80 10.1111/j.2042-7158.1993.tb03687.x 8094455

[B74] Singh A. Reed M. J. (1991). Insulin-like growth factor typeI and insulin-like growth factor type II stimulate oestradiol-17 beta hydroxysteroid dehydrogenase (reductive) activity in breast cancer cells. *J. Endocrinol.* 129 5–8. 2040851 10.1677/joe.0.129r005

[B75] Sohn E. J. Won G. Lee J. Lee S. Kim S. H. (2015). Upregulation of miRNA3195 and miRNA374b mediates the anti-angiogenic properties of melatonin in hypoxic PC-3 prostate cancer cells. *J. Cancer* 6 19–28. 10.7150/jca.9591 25553085 PMC4278911

[B76] Thomas M. Augustin H. G. (2009). The role of the angiopoietins in vascular morphogenesis. *Angiogenesis* 12 125–137. 10.1007/s10456-009-9147-3 19449109

[B77] Trenti A. Tedesco S. Boscaro C. Trevisi L. Bolego C. Cignarella A. (2018). Estrogen, angiogenesis, immunity and cell metabolism: solving the puzzle. *Int. J. Mol. Sci.* 19 859. 10.3390/ijms19030859 29543707 PMC5877720

[B78] Vannini F. Kashfi K. Nath N. (2015). The dual role of iNOS in cancer. *Redox Biol.* 6 334–343. 10.1016/j.redox.2015.08.009 26335399 PMC4565017

[B79] Viallard C. Larrivée B. (2017). Tumor angiogenesis and vascular normalization: alternative therapeutic targets. *Angiogenesis* 20 409–426. 10.1007/s10456-017-9562-9 28660302

[B80] Vijayalaxmi Reiter R. J. Tan D. X. Herman T. S. Thomas C. R. Jr. (2004). Melatonin as a radioprotective agent: a review. *Int. J. Radiat. Oncol. Biol. Phys.* 59 639–653. 10.1016/j.ijrobp.2004.02.006 15183467

[B81] Wachsberger P. Burd R. Dicker A. P. (2003). Tumor response to ionizing radiation combined with antiangiogenesis or vascular targeting agents: exploring mechanisms of interaction. *Clin. Cancer Res.* 9 1957–1971. 12796357

[B82] Wang R. X. Liu H. Xu L. Zhou R. X. (2016). Melatonin downregulates nuclear receptor RZR/RORγ expression causing growth-inhibitory and anti-angiogenesis activity in human gastric cancer cells in vitro and in vivo. *Oncol. Lett.* 12 897–903. 10.3892/ol.2016.4729 27446366 PMC4950661

[B83] Wang S. Ding Z. (2017). Fibroblast growth factor receptors in breast cancer. *Tumour Biol.* 39:1010428317698370. 10.1177/1010428317698370 28459213

[B84] Wang Z. Dabrosin C. Yin X. Fuster M. M. Arreola A. Rajhthmell W. K. (2015). Broad targeting of angiogenesis for cancer prevention and therapy. *Semin. Cancer Biol.* 35 S224–S243. 10.1016/j.semcancer.2015.01.001 25600295 PMC4737670

[B85] Yang L. Zheng J. Xu R. Zhang Y. Gu L. Dong J. (2014). Melatonin suppresses hypoxia-induced migration of HUVECs via inhibition of ERK/Rac1 activation. *Int. J. Mol. Sci.* 15 14102–14121. 10.3390/ijms150814102 25123138 PMC4159841

[B86] Yu H. Littlewood T. Bennett M. (2015). Akt isoforms in vascular disease. *Vascul. Pharmacol.* 71 57–64. 10.1016/j.vph.2015.03.003 25929188 PMC4728195

[B87] Zacchigna S. Zentilin L. Morini M. Dell’Eva R. Noonan D. M. Albini A. (2004). AAV-mediated gene transfer of tissue inhibitor of metalloproteinases-1 inhibits vascular tumor growth and angiogenesis in vivo. *Cancer Gene Ther.* 11 73–80. 10.1038/sj.cgt.7700657 14681728

[B88] Zonta Y. R. Martínez M. Camargo I. C. Domeniconi R. F. Lupi Júnior L. A. Pinheiro P. F. (2017). Melatonin reduces angiogenesis in serous papillary ovarian carcinoma of ethanol-preferring rats. *Int. J. Mol. Sci.* 18:E763. 10.3390/ijms18040763 28398226 PMC5412347

